# Discrete 2D Material Programming for 3D Shaping and Morphogenesis‐Inspired 4D Bioprinting

**DOI:** 10.1002/advs.77228

**Published:** 2026-08-21

**Authors:** Fereshteh Family, Aneela Davuluri, Athulya Martin, Kyungsuk Yum

**Affiliations:** ^1^ Department of Materials Science and Engineering University of Texas at Arlington Arlington USA

**Keywords:** 4D bioprinting, engineered living constructs, hydrogels, material programming, shape‐morphing materials

## Abstract

Programming in‐plane growth in thin sheets enables 2‐to‐3D shape transformation into doubly curved morphologies common in living organisms. Despite its morphogenesis‐inspired premise and intrinsic suitability for tissue‐like systems, translating growth‐programmed shaping into engineered living constructs remains challenging. Here, we report cell‐compatible discrete 2D material programming for growth‐driven 3D shaping and morphogenesis‐inspired 4D bioprinting. By patterning cell‐supportive microdomains within a responsive hydrogel matrix, we program in‐plane growth to prescribe target metrics. This approach enables 4D bioprinting of living constructs that autonomously transform into prescribed 3D morphologies under physiological conditions. We establish design rules that expand the programmable 3D shape space, characterize time‐dependent morphing dynamics, and demonstrate bioinspired motions. The transformed constructs maintain high cell viability and support tissue‐relevant cellular behaviors. Cell‐compatible discrete 2D material programming provides a platform for programmable morphogenesis and dynamic biofabrication, with potential relevance to hybrid living–synthetic systems, including bioinspired soft robotics, engineered tissue constructs, and cell‐based devices.

## Graphical abstract

Cell‐compatible discrete 2D material programming enables 2‐to‐3D shape transformation and morphogenesis‐inspired 4D bioprinting. By patterning cell‐supportive microdomains within a responsive hydrogel, the approach programs in‐plane growth to encode target metrics. Cell‐laden planar constructs morph into prescribed 3D geometries under physiological conditions while supporting tissue‐relevant cellular behaviors. This platform advances programmable morphogenesis and dynamic biofabrication of hybrid living–synthetic systems.

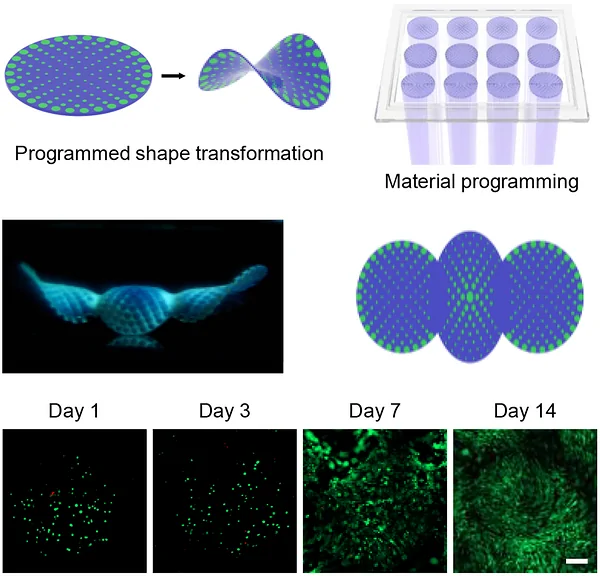

## 1 Introduction

Living organisms generate complex morphologies and motions through differential growth—spatially regulated expansion and contraction—of soft tissues [1, 2, 3, 4]. Inspired by biological morphogenesis, programming spatially controlled in‐plane growth in thin sheets drives transformation into prescribed three‐dimensional (3D) shapes [5, 6, 7, 8]. The 3D shapes emerging from thin materials are ubiquitous in both nature and engineered systems and underpin diverse functions, spanning leaves and petals, organ epithelia, batoid fins, engineered thin shells such as aircraft skins and vehicle body panels, and flexible electronic skins [1, 2, 3, 4, 5]. Such shape‐morphing (“4D”) materials enable emerging technologies, including bioinspired manufacturing [7, 8, 9], soft robotics [10, 11], reconfigurable devices [12, 13], curved microfluidics [14], and tissue engineering [15]. In growth‐driven 3D shaping, spatially controlled growth prescribes a target metric—a metric tensor that defines local distances—whose incompatibility with a planar embedding induces programmed out‐of‐plane deformation [16]. This metric programming enables doubly curved morphologies and motions with non‐zero Gaussian curvature (*K* ≠ 0), common in living organisms yet difficult to realize with conventional fabrication methods [5, 6, 7, 8, 9]. In living systems, such shapes emerge because differential growth drives metric changes during morphogenesis [1, 2, 3, 4]. By contrast, homogeneous growth in a flat sheet preserves the planar metric, so achievable shapes are restricted to isometries of a flat surface—developable geometries with *K* = 0, a constraint that underlies many biomorph‐type bending strategies [17, 18, 19].

Growth‐driven 3D shaping has been realized by programming spatially controlled expansion and contraction in responsive material systems, including hydrogels [5, 6, 7, 8, 9], liquid crystal elastomers [20], dielectric elastomers [21], and inflatable structures [22]. Among these material systems, hydrogels are particularly attractive for bioinspired and biomedical applications due to their soft‐tissue‐like physical properties and biochemical tunability [23, 24]. In this context, growth‐driven shaping offers a morphogenesis‐inspired route to complex 3D geometries via target‐metric programming, and hydrogels are appealing candidates for the biofabrication of cell‐laden constructs because they can be engineered to present tissue‐like mechanics and biochemical cues [23, 24]. However, integrating target‐metric programmability with direct cell encapsulation under physiological conditions remains challenging, as few material systems are both shape‐programmable and cell‐compatible. Many programmable materials (including stimuli‐responsive hydrogels) and associated material‐programming and shape‐morphing workflows require nonphysiological chemistries, processing conditions, or actuation environments (e.g., temperature, pH, or solvent conditions outside physiological ranges), which can preclude direct cell encapsulation or compromise cell viability and long‐term function [5, 6, 7, 8, 9, 20, 21, 22]. This challenge motivates a programming strategy that decouples actuation from the cellular microenvironment by encoding target metrics in an actuating matrix while confining cells to cell‐supportive microdomains, thereby enabling growth‐driven shaping under physiological conditions.

The 4D bioprinting involves the 2D or 3D printing of cell‐laden constructs engineered to change shape over time in response to external or cell‐generated cues [25, 26, 27, 28]. Many 4D bioprinting strategies have relied on bilayer (bimorph)‐type bending, hinge‐like actuation, or related through‐thickness strain‐gradient mechanisms [29, 30, 31, 32, 33, 34]. These mechanisms are readily implementable but often restrict achievable shapes to bimorph‐type bending geometries with near‐zero Gaussian curvature (*K* ≈ 0) [29, 30, 31, 32, 33, 34], limiting access to doubly curved morphologies prevalent in living organisms (*K* ≠ 0) [6, 7, 8, 9]. By contrast, growth‐programmed (target‐metric) shaping encodes an in‐plane growth field that prescribes a non‐Euclidean metric and has enabled programmable access to doubly curved geometries in acellular systems [5, 6, 7, 8, 9]; however, its translation to cell‐laden 4D printing remains largely unexplored. Shape design in conventional 4D bioprinting is also frequently guided by qualitative deformation modes and iterative experimental optimization, while quantitative models remain largely confined to simplified geometries such as bilayer bending [19, 33, 34]. Moreover, relatively few 4D bioprinting studies report sustained tissue‐relevant cellular behaviors within shape‐morphed constructs, including spreading, migration, multicellular assembly, and cytoskeletal organization [18, 30]. In addition, throughput and scalability are commonly constrained by serial extrusion‐based printing and the reliance on bilayer architectures [29, 30, 31, 32, 33]. These constraints can increase fabrication time and impose stringent demands on interfacial adhesion under large mismatch strains, potentially compromising cell viability and robustness.

**FIGURE 1:**
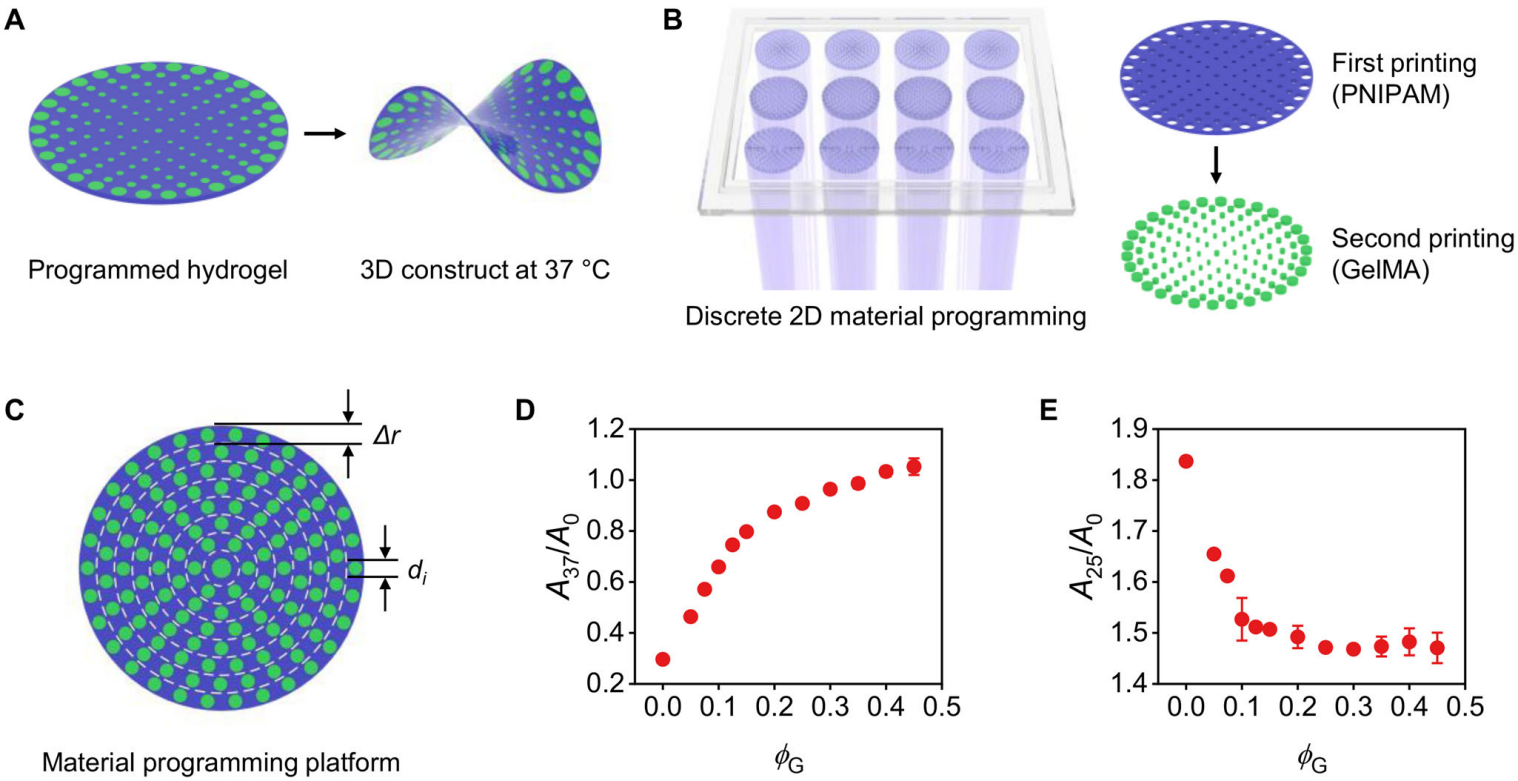
Cell‐compatible discrete 2D material programming for 3D shaping. (A) Illustration of programmed shape transformation from a planar hydrogel to a target 3D construct. (B) Discrete 2D material programming by printing composite hydrogels with spatially encoded in‐plane growth Ω (target metric) using multimaterial digital light lithography. This process enables parallel printing of multiple hydrogels with distinct target metrics. (C) Axisymmetric material programming platform comprising a central disk of radius ∆*r* containing a single GelMA circle of diameter *d*_0_, surrounded by annuli of radial thickness ∆*r*, each containing *n_i_* ​ GelMA circles of diameter *d_i_* ​. (D) Areal shrinkage ratio *A*_37_/*A*_0_ of composite hydrogel disks with uniform *d_i_* ​ as a function of GelMA area fraction *ϕ*_G_ in PBS at 37°C. *A*_37_/*A*_0_ = 1.066 ± 0.001 for the GelMA‐only condition (*ϕ*_G_ = 1). (E) Areal swelling ratio *A*_25_/*A*_0_ of composite hydrogel disks with uniform *d_i_* ​ as a function of *ϕ*_G_ in PBS at 25°C. *A*_25_/*A*_0_ = 1.123 ± 0.012 for the GelMA‐only condition (*ϕ*_G_ = 1). Data are mean ± s.d (*n* = 5 independent structures) in (D, E).

Here, we report cell‐compatible discrete 2D material programming for growth‐driven 3D shaping, enabling morphogenesis‐inspired 4D bioprinting of living constructs that transform into prescribed 3D morphologies (Figure 1). In this study, 4D bioprinting refers to 2D lithographic printing of planar constructs containing cell‐laden domains followed by material‐programmed morphing into target 3D geometries over time under physiological conditions. To address the challenge of achieving both programmable shape morphing and cell compatibility, we implement a gelatin methacryloyl (GelMA)–poly(*N*‐isopropylacrylamide) (pNIPAm) domain–matrix architecture that decouples actuation from cell support. In this architecture, the target metric is programmed by spatially controlled pNIPAm contraction while cells are confined to cell‐supportive GelMA domains, enabling direct cell encapsulation and culture under physiological conditions while retaining quantitative target‐metric programming. The pNIPAm matrix provides temperature‐triggered actuation across the room temperature‐to‐37°C transition [7], whereas GelMA domains provide microenvironments for direct cell encapsulation and sustained viability and proliferation [35, 36]. Because actuation primarily resides in the pNIPAm matrix, this architecture limits mechanical perturbation of encapsulated cells during morphing. Although pNIPAm‐based materials have been used in a range of biomedical applications [37, 38], direct cell encapsulation in pNIPAm‐based matrices under physiological conditions is generally not cell‐supportive and can reduce cell viability and proliferation [36], likely due in part to the high‐density polymer network in the shrunken state at 37°C and the absence of cell‐instructive biochemical cues [23, 39, 40]. Accordingly, direct translation of pNIPAm‐based growth‐programmed shaping [6, 7, 8] to cell‐laden 4D bioprinting is not readily achievable without a cell‐compatible strategy.

We achieve programmable 3D shaping by 2D printing composite hydrogels with a spatially encoded in‐plane growth field Ω (contraction in this study) (Figure 1A,B). This programming is implemented by patterning temperature‐unresponsive, cell‐compatible GelMA domains within a temperature‐responsive pNIPAm matrix using multimaterial digital light lithography. The resulting domain pattern locally suppresses pNIPAm contraction, encoding the target metric. Upon exposure to aqueous media at 37°C, the printed constructs spontaneously morph into target 3D geometries. Building on this framework, we establish design rules—based on integration, transformation, and modular assembly of target metrics—that further expand the programmable 3D shape space. We characterize time‐dependent morphing dynamics and demonstrate bioinspired motions. The domain–matrix architecture enables direct encapsulation of living cells within GelMA domains while maintaining high cell viability during material programming and subsequent shape transformation. The transformed constructs support tissue‐relevant cellular behaviors, including spreading, migration, multicellular assembly, and organized cytoskeletal architecture. Moreover, the parallel 2D printing of multiple constructs with individualized shape programs enables scalable and customizable 4D bioprinting (Figure 1B). Together, these capabilities establish cell‐compatible discrete 2D material programming as a platform for programmable morphogenesis and dynamic biofabrication, expanding the accessible shape space of 4D bioprinting toward doubly curved geometries with non‐zero Gaussian curvature (*K* ≠ 0).

## 2 Results

### 2.1 Cell‐Compatible Discrete 2D Material Programming for 3D Shaping

To program 2D hydrogels with spatially controlled growth for 3D shaping under physiological conditions, we developed a cell‐compatible discrete 2D material programming strategy that patterns temperature‐unresponsive GelMA domains within a temperature‐responsive pNIPAm matrix using multimaterial digital light lithography (Figure 1). This domain–matrix architecture decouples actuation from cell support, enabling growth‐driven shaping under physiological conditions. We selected GelMA as a cell‐supportive phase because it retains extracellular matrix‐derived bioactivity and enables the formation of cell‐instructive hydrogels that support cell encapsulation and culture under our printing and shape‐morphing conditions [23, 24, 36]. By controlling the area fraction of GelMA domains, we modulated the local effective contraction of the composite hydrogel at 37°C, thereby encoding the growth field Ω. A key requirement for reliable metric programming is that the GelMA domains are sufficiently stiff to constrain the local matrix contraction—yet sufficiently soft to support cell viability and function. In our system, 5 wt.% GelMA forms a mechanically stable phase (elastic modulus *E* = ∼1.8 kPa) that effectively suppresses the local pNIPAm contraction while maintaining a soft, cell‐supportive microenvironment for encapsulated cells [39, 40]. This two‐material patterning strategy provides a discrete alternative to continuous‐tone programming via grayscale lithography [7, 8] while allowing the direct encapsulation of living cells within GelMA domains, establishing a cell‐compatible route to 3D shaping. Moreover, compared with continuous‐tone programming, which relies on precise control of intermediate crosslinking to modulate local swelling or shrinkage behavior [7, 8], discrete programming relaxes this dose‐control requirement, but at the cost of reduced effective programming resolution.

To implement discrete 2D material programming for 3D shaping, we designed a disk‐shaped platform composed of concentric regions (Figure 1C and Figure S1). This platform comprises a central disk of radius ∆*r* containing a single GelMA circle of diameter *d*_0_, surrounded by annuli of radial thickness ∆*r*, each containing *n_i_* ​ GelMA circles of diameter *d_i_* ​, where *i* indexes the annulus from the center outward. We determined *n_i_* ​ to (i) maintain a constant GelMA area fraction *ϕ*_G_ across annuli for a given *d*_i_ and (ii) satisfy *ϕ*_G_ = 0.5 when *d*_i_ = ∆*r*, as shrinkage control saturated near *ϕ*_G_ = 0.5. This design enables local control of *ϕ*_G_ by varying *d*_i_, decoupling area‐fraction modulation from the number of GelMA circles per annulus and thereby facilitating the translation of a prescribed growth field Ω (target metric) into a discrete format. To further promote uniform spatial distribution, the angular positions of GelMA circles are alternated between adjacent annuli by applying an angular offset of 2π/*n*_i_​ to every other annulus (Figure S1). This modular, axisymmetric platform provides a generalizable geometric architecture for discrete 2D material programming.

To program 2D hydrogels with Ω by controlling local *ϕ*_G_, we established a quantitative relationship between areal shrinkage (and swelling) and *ϕ*_G_ using composite hydrogel disks with uniform *d*_i_ (Figure 1D,E). These disks exhibited globally homogeneous shrinkage and swelling behaviors that varied with *ϕ*_G_ in phosphate‐buffered saline (PBS) at 37°C and 25°C, respectively. The areal shrinkage ratio *A*_37_/*A*_0_ at 37°C increased with *ϕ*_G_, ranging from Ω_min_ = 0.3 at *ϕ*_G_ = 0 to Ω_max_ = 1.05 at *ϕ*_G_ = 0.45 (Figure 1D). Conversely, the areal swelling ratio *A*_25_/*A*_0_ at 25°C decreased with *ϕ*_G_ (Figure 1E). These results indicate that the GelMA domains constrain both the shrinkage and swelling of the pNIPAm matrix. These relationships enable the translation of continuous Ω into discrete material patterns by locally controlling *ϕ*_G_, allowing shape programming in either the shrunken or swollen state. To realize 3D shaping under physiological conditions, we designed target shapes in the shrunken state (37°C).

**FIGURE 2:**
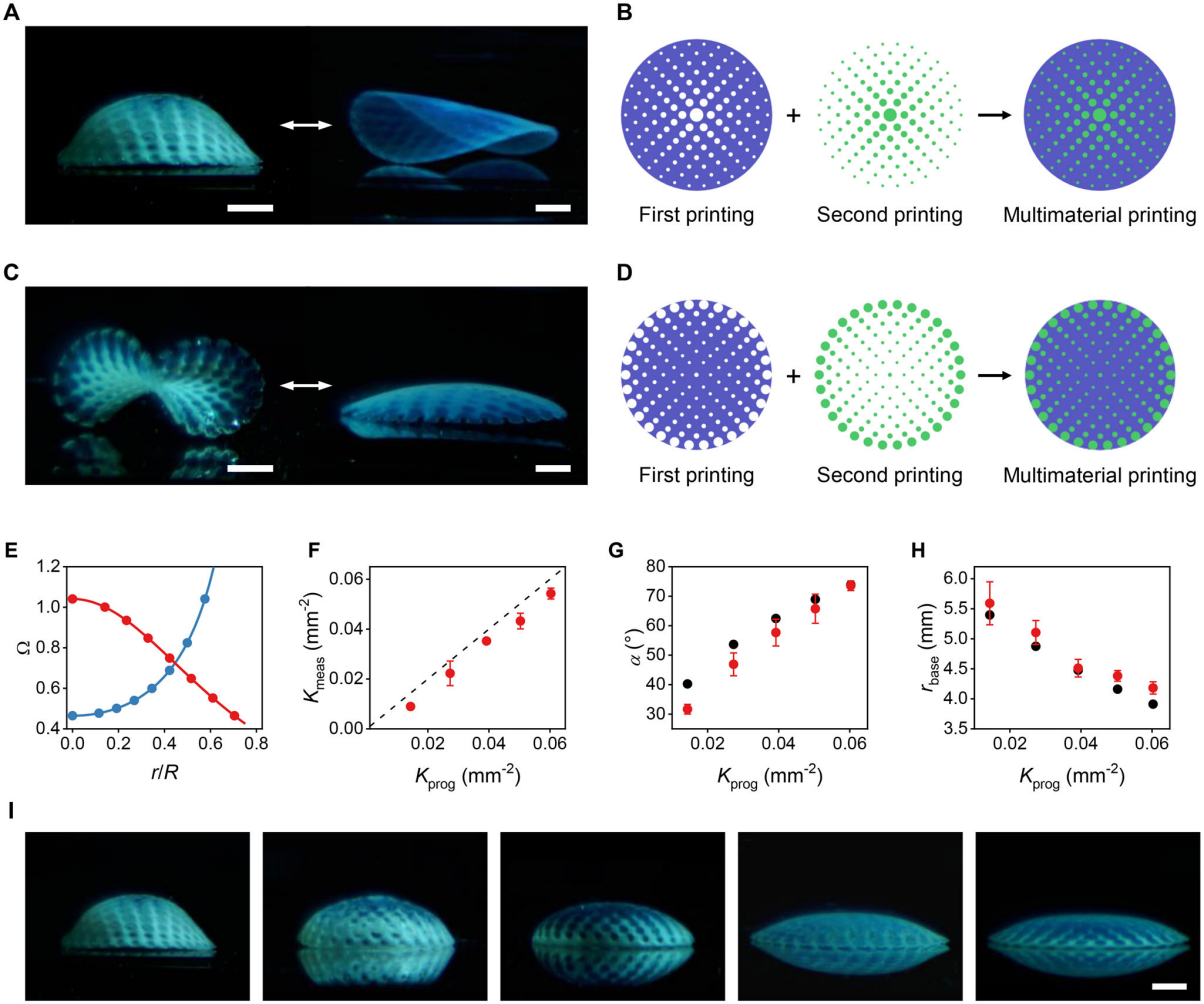
Growth‐driven 3D shaping. (A) Formation of a spherical‐cap structure with constant positive Gaussian curvature (*K* > 0). (B) Multimaterial printing pattern used to form the spherical‐cap structure in (A) (right); left: first (pNIPAm) and second (GelMA) printing patterns. (C) Formation of a saddle structure with constant negative Gaussian curvature (*K* < 0). (D) Multimaterial printing pattern used to form the saddle structure in (C) (right); left: first (pNIPAm) and second (GelMA) printing patterns. (E) Ω(*r*/*R*) used to program spherical‐cap (red) and saddle (blue) structures in (A–D). Solid circles indicate discretized Ω values used for discrete material programming. (F) Measured Gaussian curvature *K*_meas_ of spherical‐cap structures programmed with target Gaussian curvature *K*_prog_ of 0.014, 0.027, 0.039, 0.050, and 0.060 mm^−2^ (red circles). The dashed line indicates the theoretical *K*. (G) Measured (red circles) and theoretical (black circles) cap angle (polar angle from apex to base) *α* vs. *K*_prog_. (H) Measured (red circles) and theoretical (black circles) base radius *r*_base_ vs. *K*_prog_. (I) Representative spherical‐cap structures programmed with *K*_prog_ of 0.060, 0.050, 0.039, 0.027, and 0.014 mm^−2^ (from left to right). Data are mean ± s.d (*n* = 3 independent structures) in (F–H). Scale bars, 2 mm (left) and 3 mm (right) (A and C); 2 mm (I).

Having established discrete control over shrinkage using homogeneous composite disks, we next validated our discrete 2D material programming approach by printing geometric 3D structures: spherical‐cap and saddle shapes with constant positive and negative Gaussian curvature (*K* > 0 and *K* < 0), respectively (Figure 2). Our 2D‐to‐3D shaping strategy is based on two fundamental principles [5, 7, 16]. First, for thin elastic sheets, bending is energetically favored over stretching (bending energy *E*_b_ ∼ *t* ^3^ whereas stretching energy *E*_s_ ∼ *t*, where *t* is the sheet thickness). Differential in‐plane growth is therefore accommodated predominantly by out‐of‐plane deformation with only a small amount of in‐plane strain. Second, the 3D configuration is an embedding of the target metric prescribed by Ω. By Gauss's *theorema egregium*, Ω determines the local Gaussian curvature, *K* = −∆(ln Ω)/(2Ω), where ∆ is the Laplacian [41]. As *t* → 0, the 3D shape converges to a stretch‐free, low‐bending‐energy isometric embedding (target 3D shape). Accordingly, increasing the ratio of thickness to a characteristic in‐plane length scale (*t*/*L*) is expected to increase deviations from the target‐metric embedding; conversely, maintaining a small *t*/*L* (or *t* → 0) preserves bending‐dominated accommodation of the programmed metric, improving shape fidelity as *t*/*L* → 0 and enabling application across a range of length scales [7, 8, 42]. In our domain–matrix design, out‐of‐plane deformation is primarily driven by the differential contraction of the pNIPAm matrix, whereas the GelMA microdomains are expected to remain near strain‐free, experiencing only modest interfacial loading.

We created spherical cap and saddle structures (Figure 2 A,C and Figure S2) by programming hydrogels with Ω (Figure 2B,D), defined as Ω(*r*) = *c*[1 + (*r*/*R*)^2^]^−2^ for spherical caps and Ω(*r*) = *c*[1 − (*r*/*R*)^2^]^−2^ for saddles, where *r* is the radial coordinate, and *c* and *R* are constants (Figure 2E) [7]. To implement Ω in our axisymmetric platform (Figure 1C), we discretized Ω(*r*) (Figure 2E) into spatially patterned GelMA domains via multimaterial printing (Figure 2B,D) [8]. Specifically, we evaluated Ω(*r*) at the center (*r* = *r*_0_ = 0) and the midpoint of each annulus (*r* = *r*_i_ = (*i* + 0.5)∆*r*), computed the corresponding *ϕ*_G_ using the calibration curve (Figure 1D), and then determined the diameters of the GelMA circles in the central and annular regions (*d*_0_ and *d_i_* ​, respectively). These structures reversibly transformed between the shrunken and swollen states in response to temperature changes (Figure 2A,C and Movies S1 and S2).

The printed structures quantitatively agreed with theoretical models, supporting shape programmability (Figure 2F–I). The experimentally measured *K* values of the spherical‐cap and saddle structures in Figure 2A,C (0.056 and −0.092 mm^−2^, respectively) closely matched the corresponding programmed values (0.060 and −0.090 mm^−2^). To further evaluate shape programmability, we printed spherical‐cap and saddle structures spanning a range of programmed *K* values (Figure 2I and Figure S3). The measured *K* values (*K*_meas_) closely followed the programmed values (*K*_prog_), demonstrating geometric control over 3D shape formation via discrete material programming (Figure 2F and Figure S4). However, the saddle structures showed a principal‐curvature asymmetry (*k*_1_ ≠ −*k*_2_), likely due to substrate interactions during shape transformation. We further quantified key global geometric metrics of the spherical‐cap structures—including cap angle (polar angle from apex to base), base radius, and radius of curvature—and observed close agreement with theoretical values (Figure 2G,H and Figure S4), demonstrating high shape fidelity.

To examine whether this programming principle extends beyond the GelMA–pNIPAm material pair, we tested methacrylated hyaluronic acid (HAMA) and poly(ethylene glycol) diacrylate (PEGDA) as alternative domain materials within pNIPAm matrices (Figures S5 and S6). HAMA and PEGDA were selected because of their established use in tissue engineering and biomedical applications [23, 24]. Using both HAMA–pNIPAm and PEGDA–pNIPAm systems, we programmed spherical‐cap and saddle constructs as representative target shapes with positive and negative Gaussian curvature, respectively. Both material systems formed the prescribed positive‐ and negative‐curvature geometries, supporting the broader applicability of the domain–matrix programming strategy. Together, these results validate a quantitative route for discretizing target metrics into cell‐compatible material patterns and support the material extensibility of the domain–matrix programming strategy.

### 2.2 Design Rules for Complex 3D Constructs

**FIGURE 3:**
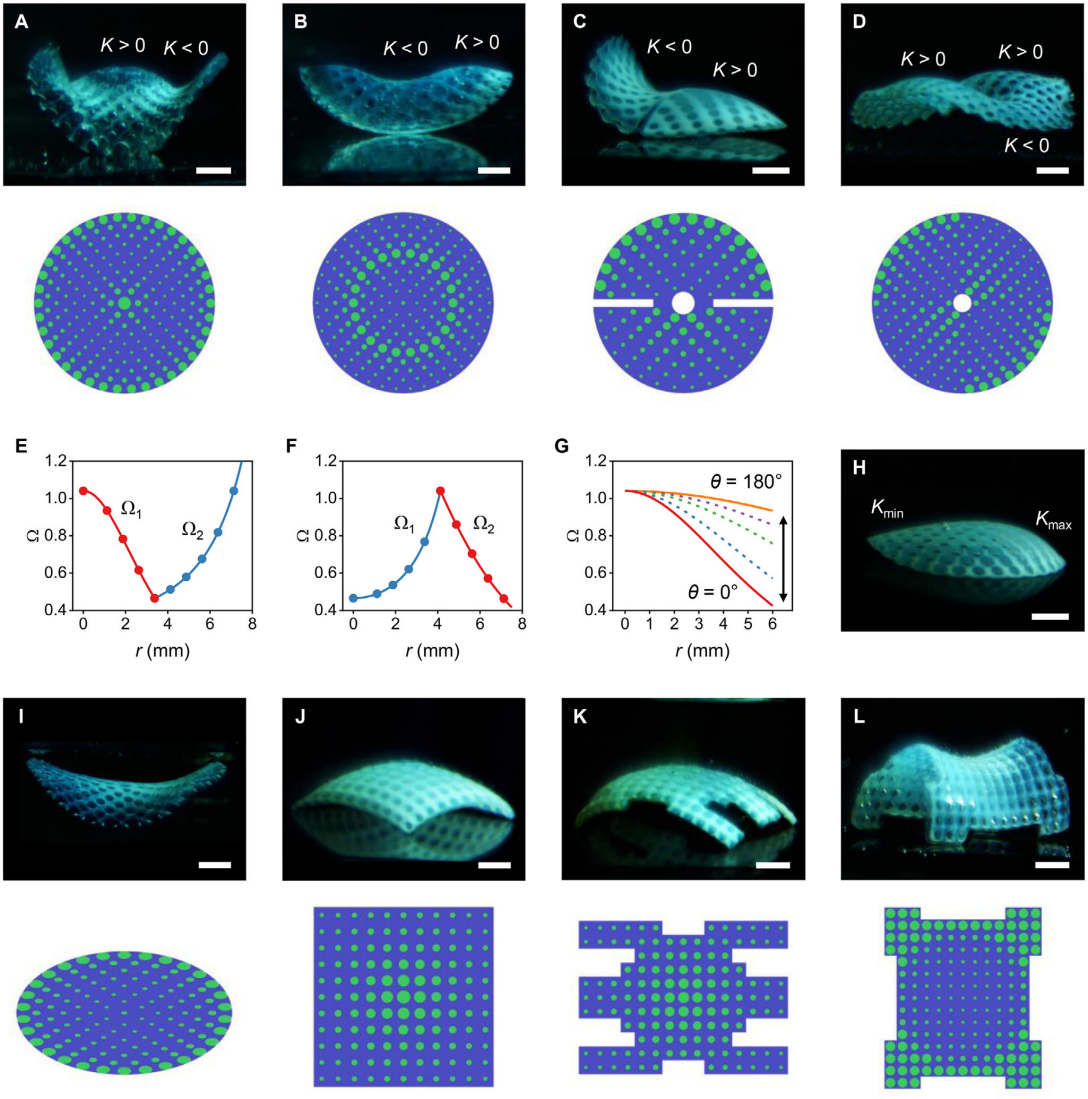
Design rules for complex 3D constructs. (A–F) Integration of target metrics. Hybrid constructs generated by radial (A, B) and azimuthal (C, D) integration of target metrics Ω (top) with the corresponding printing patterns used for material programming (bottom). Hybrid Ω functions used to program the constructs shown in (A, B) are shown in (E, F), respectively. (G, H) A construct exhibiting smoothly varying *K* along *θ* (H), programmed using Ω(*r*, *θ*) that varies smoothly with *θ* (G). (I) Elongated saddle construct (top) generated by an elliptically elongated printing pattern (bottom). (J–L) Constructs with programmable boundary geometries (top) and the corresponding printing patterns used for material programming (bottom). Scale bars, 2 mm (A–D, H, and J–L); 3 mm (I).

**FIGURE 4:**
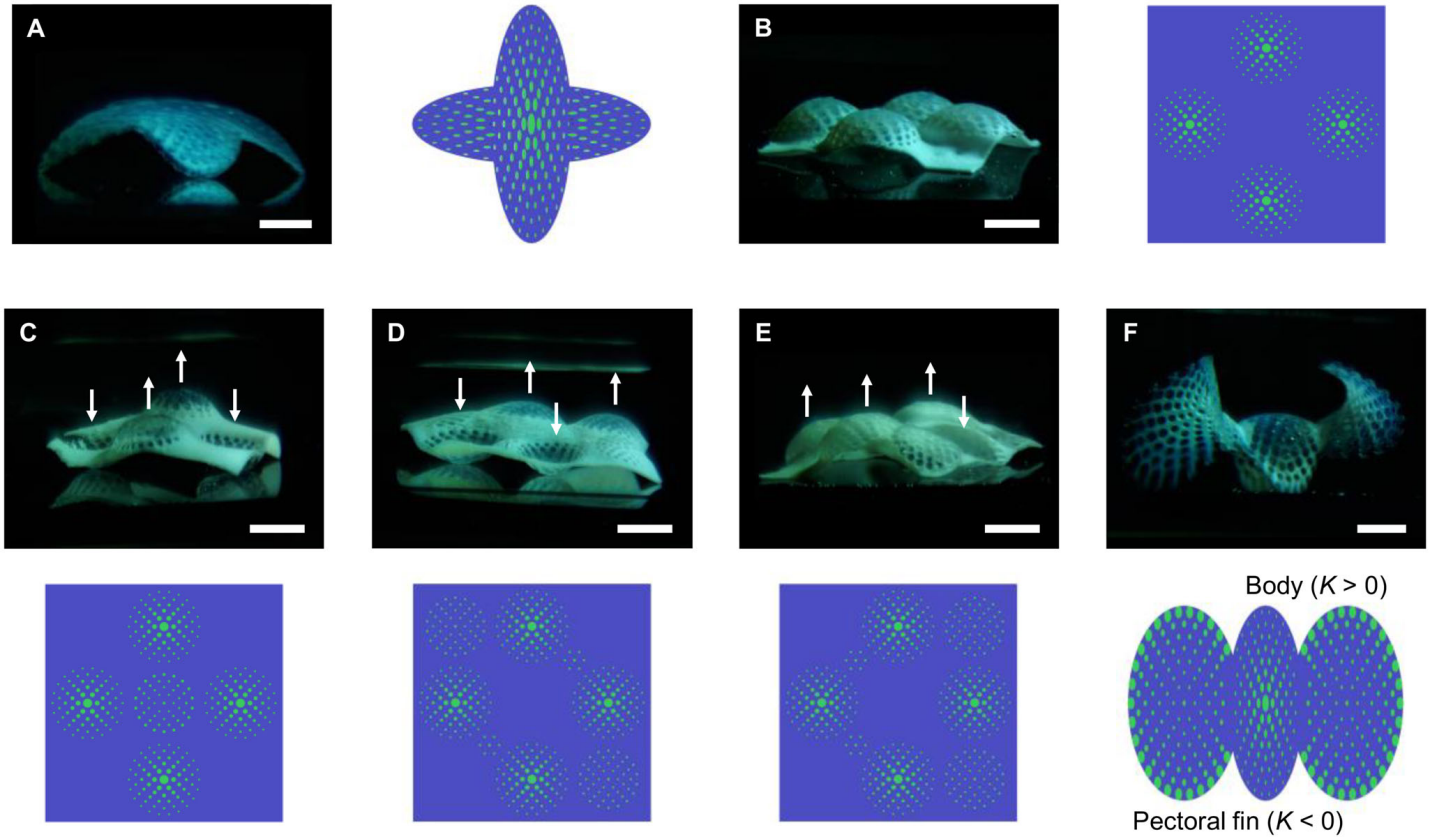
Multimodular 3D constructs enabled by modular assembly and shape guiding. (A) Spherical‐cap‐like construct with four leg‐like extensions formed by assembling two elongated spherical‐cap modules (left) and the corresponding printing pattern used for material programming (right). (B–E) Four‐spherical‐cap assemblies without shape‐guiding modules (B) and with shape‐guiding modules (C–E), together with the corresponding printing patterns used for material programming. White arrows indicate the programmed upward and downward morphing directions of cap domains. (F) Stingray‐inspired construct comprising a central body module (*K* > 0) and pectoral fin modules (*K* < 0) (top) and the corresponding printing pattern used for material programming (bottom). Scale bars, 3 mm (A and F); 5 mm (B–E).

Building on the validation of discrete material programming with axisymmetric target metrics, we established design rules for generating 3D constructs with diverse morphologies (Figures 3 and 4). These rules comprise three strategies—integration, transformation, and modular assembly of target metrics—that collectively provide a quantitative framework for expanding the programmable 3D shape space.

Integration of target metrics along radial or azimuthal coordinates produces hybrid 3D constructs with regions of distinct Gaussian curvature (Figure 3A–F and Figures S7–S11). Radial integration of Ω_1_ for a spherical cap (*i* = 0–5) and Ω_2_ for a saddle (*i* = 5–10) yielded a construct with *K* > 0 in the central region and *K* < 0 in the periphery (Figure 3A,E and Figure S7). Conversely, radial integration of Ω_1_ for a saddle (*i* = 0–6) and Ω_2_ for a spherical cap (*i* = 6–10) generated a reversed curvature profile, with central *K* < 0 and peripheral *K* > 0 regions (Figure 3B,F). Likewise, azimuthal integration of Ω_1_ (0< *θ* < π) and Ω_2_ (π < *θ* < 2π) produced a construct with alternating curvature domains around the circumference, where *θ* is the angular coordinate (Figure 3C and Figures S8–S10). Because sharp discontinuities in Ω at metric interfaces can lead to stress accumulation and morphological distortion during shape morphing [6, 7, 8], we engineered these boundaries using cuts and linkers (Figure 3C,D and Figures S8–S11). Cuts reduced interfacial interference and improved the definition of curvature domains (Figure S9 and Table S1). Linkers—created by resizing GelMA circles at the interface to the average diameter of circles in Ω_1_ and Ω_2_ within the corresponding annulus—provided smoother metric continuity and yielded slightly better‐defined curvature domains than constructs without linkers (Figure S9 and Table S1). Moreover, azimuthal integration of Ω_1_ (0< *θ* < π/2 and π < *θ* < 3π/2) and Ω_2_ (π/2< *θ* < π and 3π/2< *θ* < 2π) with linkers produced a four‐domain construct featuring alternating *K* > 0 and *K* < 0 regions (Figure 3D and Figure S11).

Transformation of axisymmetric target metrics into nonaxisymmetric forms generates nonaxisymmetric constructs with diverse morphologies (Figure 3G–L and Figures S12–S15). One transformation strategy introduces azimuthal modulation into axisymmetric Ω, yielding nonaxisymmetric constructs whose morphologies vary continuously along *θ* (Figure 3G,H). For example, transforming Ω(*r*) for a spherical cap into Ω(*r*, *θ*) = Ω(*r*/(*a*(*θ*)*R*)) = *c*[1 + (*r*/(*a*(*θ*)*R*))^2^]^−2^ with *a*(*θ*) = [1 + *b* sin^2^(*θ*/2)]^1/2^ prescribes a nonaxisymmetric shape with smoothly varying *K*, in which the curvature decreases continuously from a maximum at *θ* = 0 to a minimum at *θ* = π and then increases back to the maximum at *θ* = 2π, where *b* > 0 is a constant. Experimentally, implementing Ω(*r*/(*a*(*θ*)*R*)) with *b* = 9.2 (Figure 3G and Figure S12) yielded a construct that exhibited the predicted smoothly varying curvature profile, spanning *K*_max_ = 0.057 mm^−2^ to *K*_min_ = 0.014 mm^−2^ (Figure 3H).

A second transformation strategy deforms target metrics into elongated forms, prescribing shapes whose boundaries vary along *θ* while preserving the functional form and thus the morphology along *r* (Figure 3I and Figures S13 and S14). We realized this transformation by deforming axisymmetric printing patterns into elliptical ones, producing elongated spherical‐cap (Figure S13) and saddle (Figure 3I and Figure S14) constructs. In elongated saddle constructs, the principal curvature directions at the center aligned with the ellipse's major and minor axes [7, 8].

A third transformation strategy extends the axisymmetric programming framework by recasting Ω(*r*, *θ*) into Ω(*x*, *y*), enabling constructs with programmable boundary geometries and diverse morphologies (Figure 3J–L and Figure S15). To validate this strategy, we generated Ω(*x*, *y*) from Ω(*r*) and produced spherical‐cap and saddle constructs with square boundaries (Figure 3J and Figure S15). We translated Ω(*x*, *y*) into printing patterns by remapping the positions and sizes of GelMA circles from polar (*r*, *θ*) to Cartesian (*x*, *y*) coordinates, assigning each pixel a prescribed GelMA area fraction *ϕ*_G_. This transformation further expanded the shape space to include constructs with arbitrarily programmable boundary geometries (Figure 3J–L and Figure S15).

Modular assembly of target metrics enables complex 3D constructs with integrated geometries and functionalities (Figure 4). Assembling two elongated spherical‐cap modules produced a spherical‐cap‐like construct with four leg‐like extensions (Figure 4A). Combining four spherical‐cap modules yielded a construct with four spherical‐cap domains (Figure 4B). Because a target metric prescribes Gaussian curvature *K* but does not, in general, uniquely determine the configuration, multimodular metrics can admit multiple isometric embeddings, often with reflection‐related degeneracy [7, 8]. To actively coordinate shape selection in modular assemblies, we adopted shape‐guiding modules—localized metric features with small |*K*| that couple the morphing directions of adjacent regions while minimally perturbing the global target shape (Supporting Information) [7, 8]. In four‐spherical‐cap assemblies, shape‐guiding modules directed each cap domain to deform in its programmed upward or downward direction (Figure 4C–E), whereas an unguided assembly yielded a construct in which all cap domains deformed in a single direction (Figure 4B).

Combining these principles, we created stingray‐inspired constructs that reproduce the key morphological features of stingrays: a central body with *K* > 0 and pectoral fins with *K* < 0 (Figure 4F and Movies S3–S6). An elongated spherical‐cap module defines the central body, while two elongated saddle modules form the pectoral fins. The central body functions as a shape‐guiding module that synchronizes the morphing of the fins: along the body–fin interfaces, the fins share the body's longitudinal principal‐curvature direction, coordinating left and right fins to deform coherently.

### 2.3 Dynamic Shape Morphing of 2D‐Programmed 3D Constructs

**FIGURE 5:**
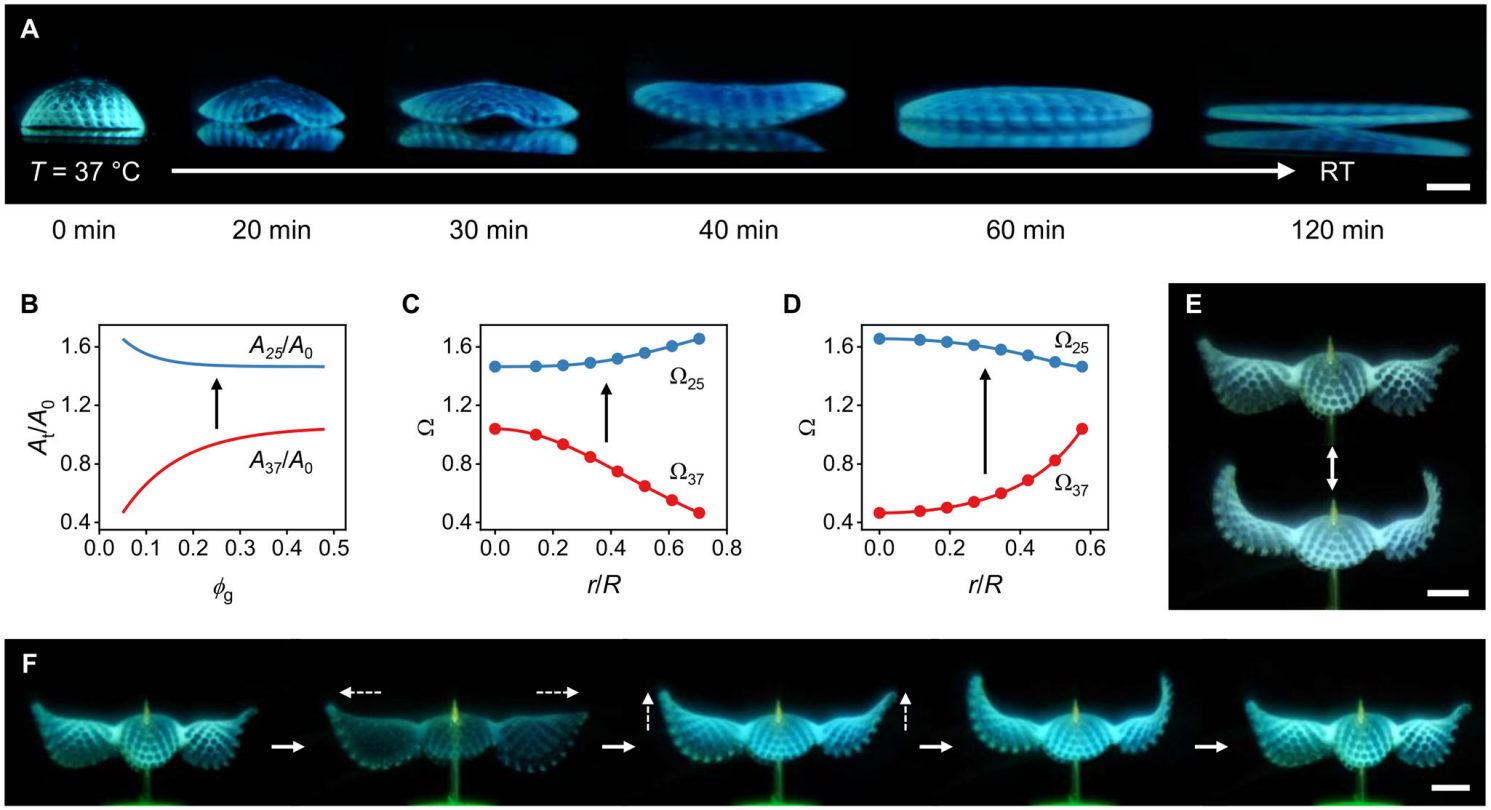
Dynamic shape morphing and bioinspired motion of 2D‐programmed 3D constructs. (A) Shape evolution of a spherical‐cap structure during cooling from 37°C to room temperature (RT). (B) Static equilibrium calibration curves at 37°C (*A*_37_/*A*_0_, red) and RT (*A*_25_/*A*_0_, blue); arrow indicates the direction of transition during cooling. (C) Target metric Ω for the spherical‐cap structure in the shrunken equilibrium state at 37°C (Ω_37_, red) and the corresponding target metric in the swollen equilibrium state at RT (Ω_25_, blue); arrow indicates the direction of transition during cooling. (D) Target metric Ω for the saddle structure in the shrunken equilibrium state at 37°C (Ω_37_, red) and the corresponding target metric in the swollen equilibrium state at RT (Ω_25_, blue); arrow indicates the transition direction during cooling. (E) Synchronized, oscillatory flapping motion of a stingray‐inspired construct controlled by cyclic temperature modulation (28.5°C–34°C). (F) Shape evolution of the stingray‐inspired construct over one flapping cycle. Scale bars, 3 mm.

We investigated temperature‐responsive shape morphing of 2D‐programmed 3D constructs (Figure 5 and Figures S16–S20). Upon cooling from 37°C to room temperature, the spherical‐cap structure reversibly transformed into a complementary swollen configuration (Figure 5A and Figure S17 and Movie S1). During the transition, the construct underwent complex, time‐dependent morphing, progressing through a series of intermediate morphologies, including hybrid shapes with a cap‐like center and wrinkled periphery (e.g., at 20 and 30 min). The saddle structure also exhibited reversible shape transformations, evolving through intermediate morphologies (Figure S16 and Movie S2). Because areal shrinking and swelling vary inversely with *ϕ*_G_ (Figure 1D,E), constructs with *K* > 0 typically evolve into configurations with *K* < 0, and vice versa. The transformations were repeatable over multiple swelling–shrinking cycles (Figure S18). However, because pNIPAm‐based systems may experience fatigue or interfacial degradation under extended cycling, high‐cycle lifetime characterization will be critical for applications that require repeated shape transformations.

The intermediate morphologies arise from the continuous evolution of the underlying metrics (Figure 5B–D). As the temperature decreases toward and below the volume phase transition temperature of pNIPAm (∼32.5°C), constructs programmed with Ω in the shrunken state swell differentially, adopting time‐dependent metrics Ω_t_ and corresponding transient morphologies [7]. The dynamic metrics Ω_t_ are determined by the initial metric Ω and the evolving relationship between the areal shrinking (or swelling) ratio *A*_t_/*A*_0_ and the GelMA fraction *ϕ*_G_, referred to as the dynamic calibration curve *A*_t_/*A*_0_(*ϕ*_G_, *t*) [7]. This curve transitions from the shrunken equilibrium curve at 37°C (*A*_37_/*A*_0_, Figure 1D; *A*_t_/*A*_0_(*ϕ*_G_, 0)) to the swollen equilibrium curve at room temperature (*A*_25_/*A*_0_, Figure 1E) (Figure 5B). The evolving *A*_t_/*A*_0_(*ϕ*_G_, *t*) thereby defines Ω_t_, which transforms from Ω_37_ to Ω_25_ (Figure 5C,D), and prescribes the time‐dependent morphological evolution (Figure 5A and Figure S16).

To demonstrate the potential of our approach for bioinspired dynamic systems, we actuated a stingray‐inspired construct by applying cyclic temperature modulation (28.5°C–34°C) (Figure 5E,F and Figures S19 and S20 and Movie S7). The flapping is driven by cyclic curvature changes of the central body, which act as the primary actuating mechanism that induces and synchronizes the oscillatory motion of the left and right pectoral fins (Figure 5F). During cyclic temperature modulation, a decrease in temperature reduces the body's curvature along both longitudinal and transverse directions, extending the fins outward. A subsequent increase in temperature gradually increases the body's curvature as the system approaches the transition temperature (∼32.5°C). As the curvature begins to increase (∼31°C), a snap‐through instability is triggered, flapping the fins upward, followed by a downward motion and full restoration of the construct to its original configuration [43]. The stingray construct exhibited flapping motion over multiple cycles (Figure S20 and Movie S7). This bioinspired motion demonstrates that our approach can be extended beyond static shape morphing to enable dynamic, functionally reconfigurable systems.

### 2.4 4D Bioprinting With Cell‐Compatible Discrete 2D Material Programming for 3D Shaping

**FIGURE 6:**
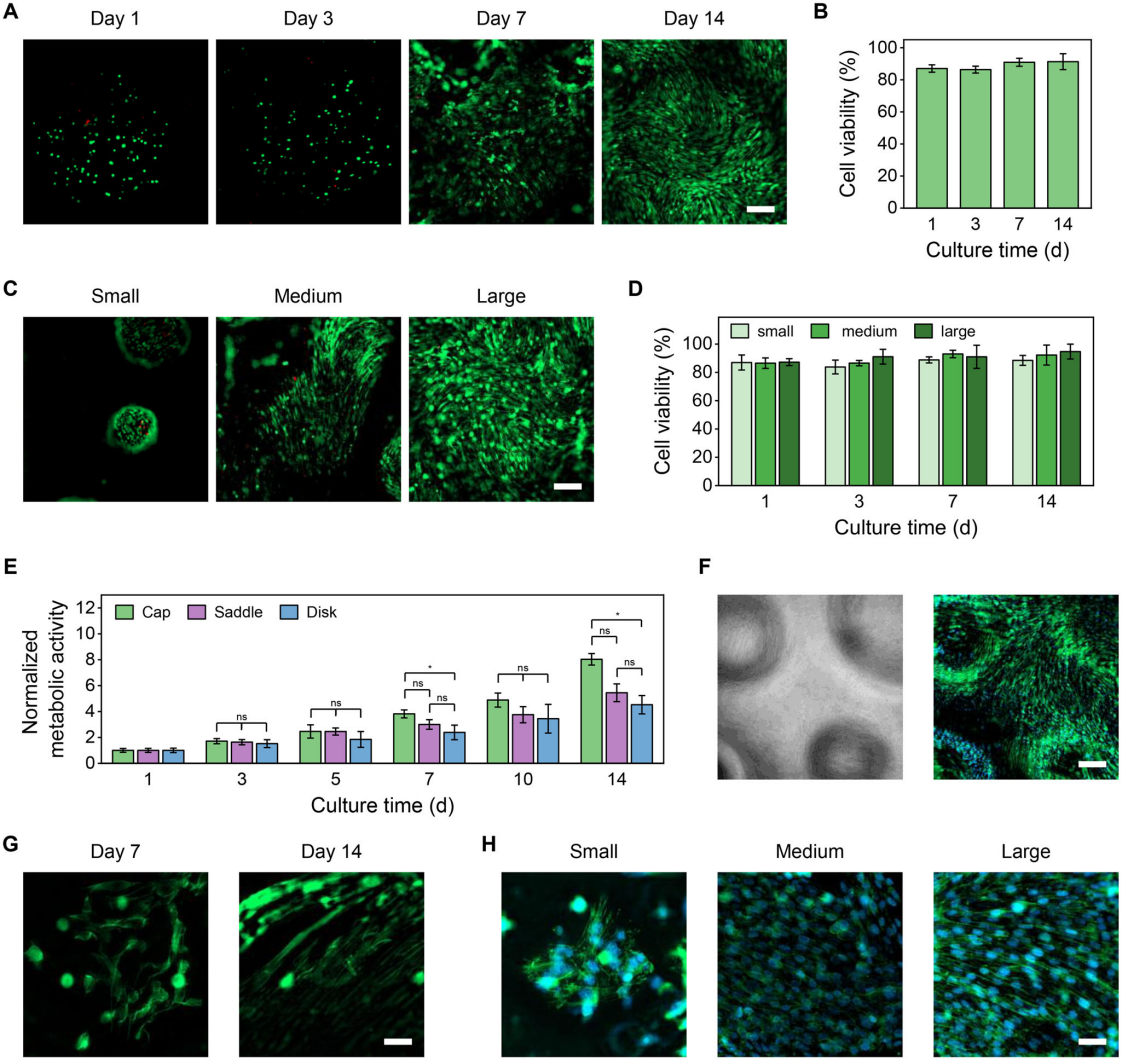
4D bioprinting of cell‐encapsulated constructs. (A) Representative fluorescence images of fibroblasts in and around GelMA domains (*ϕ*_G_ = 0.5) of spherical‐cap constructs on days 1, 3, 7, and 14 (different constructs). (B) Viability of fibroblasts in spherical‐cap constructs (*n* = 4 independent constructs at each time point). (C) Representative fluorescence images of fibroblasts in and around small, medium, and large GelMA domains of spherical‐cap constructs on day 14. (D) Viability of fibroblasts in small (*ϕ*_G_ < 0.15), medium (0.15 < *ϕ*_G_ < 0.4), and large (*ϕ*_G_ > 0.4) GelMA domains of spherical‐cap constructs. No statistically significant effect of domain size or day × size interaction was detected (two‐way ANOVA; size, *p* = 0.07; day × size, *p* = 0.70; *n* = 4 independent constructs at each time point). (E) Normalized metabolic activity of fibroblasts in spherical‐cap constructs, saddle constructs, and non‐morphing planar disk controls comprising a cell‐laden GelMA domain embedded in a pNIPAm matrix. Values were normalized to the mean metabolic activity of the corresponding construct type on day 1. Statistics: two‐way repeated‐measures ANOVA with Tukey's multiple‐comparisons test; *n* = 4 independent constructs per group, measured repeatedly over time; *p < 0.05; ns, not significant. (F) Bright field (left) and fluorescence (right) images of a spherical‐cap construct on day 14 showing cell migration out of GelMA domains. (G) Phalloidin‐stained F‐actin (green) in spherical‐cap constructs on days 7 and 14. (H) F‐actin (phalloidin, green) and nuclei (Hoechst 33342, blue) in small, medium, and large GelMA domains of spherical‐cap constructs on day 14. In (A) and (C), live cells are shown in green and dead cells in red (Live/Dead assay). In (F), live cells are shown in green, and nuclei are shown in blue. For (B, D, and E), data are mean ± s.d.; *n* values are indicated in the panel descriptions. Scale bars, 200 µm (A, C, and F); 50 µm (G, H).

By integrating cell‐compatible discrete 2D material programming with cell‐encapsulated hydrogel bioinks, we achieved 4D bioprinting of living constructs that transform into programmed 3D shapes under physiological conditions (Figure 6). We printed planar hydrogels encoded with Ω by patterning cell‐encapsulated GelMA domains within a pNIPAm matrix (Figure 1). Upon incubation in culture medium at 37°C, the planar constructs transformed into prescribed 3D geometries while maintaining high cell viability. The transformed constructs showed shape fidelity comparable to that of the cell‐free counterparts and maintained structural integrity for up to 15 days (Figure S21). The out‐of‐plane deformation is primarily accommodated by the differential contraction of the pNIPAm matrix, whereas the GelMA domains are non‐actuating; accordingly, encapsulated cells are expected to experience only modest interfacial loading during shape transformation.

To assess the cytocompatibility of our multimaterial printing process and the ensuing shape transformation, we quantified cell viability and proliferation (metabolic activity) in 4D‐bioprinted constructs (Figure 6A–E and Figures S22–S28). We fabricated cell‐laden constructs by patterning a 5 wt.% GelMA bioink encapsulating fibroblasts (2 × 10^6^ cells mL^−1^) within a pNIPAm matrix. Viability in spherical‐cap constructs was high at 87.1% on day 1 and increased to 91.3% by day 14 (Figure 6A,B and Figures S22 and S23). Viability remained high across GelMA domain sizes, with no statistically significant differences among sizes (Figure 6C,D). Viability on day 14 was comparable between spherical‐cap constructs and non‐morphing planar disk controls with a cell‐laden GelMA domain embedded in a pNIPAm matrix (Figure S24). Relative to planar GelMA‐only disk controls (printed without a pNIPAm matrix), spherical‐cap constructs showed lower viability on day 1 (87.1% vs. 97.4%) but increased by day 7 (90.9%) to a level comparable to the controls (94.3%) (Figure S25). Similarly, saddle and stingray‐inspired constructs showed high viability on day 14, reaching 96.8% and 94.4%, respectively (Figures S24, S26 and S27).

Metabolic activity increased over the 14‐day culture period, indicating sustained cellular activity after printing and shape transformation (Figure 6E and Figure S28). This increase was consistent with the increase in cell number observed by Hoechst 33342 staining (Figures S22 and S23). Normalized metabolic activity was largely comparable among spherical‐cap constructs, saddle constructs, and non‐morphing planar disk controls, with no significant differences on days 3, 5, and 10. Spherical‐cap constructs showed a greater increase in metabolic activity than disk controls on days 7 and 14, whereas the other pairwise comparisons were not significant. These results indicate that programmed shape transformation does not compromise cellular metabolic activity. The greater increase in metabolic activity observed in spherical‐cap constructs compared with static controls requires further investigation to determine whether construct geometry contributes to this difference.

Cells initially exhibited rounded morphologies and were uniformly distributed within GelMA domains (days 1 and 3), then progressively filled the domains, adopted spread morphologies, and formed multicellular assemblies by day 7 (Figure 6A). These features were more pronounced, and cells displayed predominantly elongated morphologies by day 14 (Figure 6A,C and Figure S29). Elongated cells showed locally aligned organization that was evident by day 14—including curved alignment, particularly in larger GelMA domains—consistent with mechanosensing of the circular domain geometry and coordinated migration mediated by cell–cell interactions [44]. Moreover, cells in some GelMA domains migrated outward by day 14, forming aligned multicellular structures that connected adjacent domains (Figure 6F). These observations indicate cell outgrowth beyond the initially patterned GelMA domains; however, the fluorescence images do not unambiguously distinguish bulk migration within the pNIPAm matrix from surface‐associated migration along the matrix surface. Collectively, these results indicate active cell–matrix remodeling and the emergence of coordinated multicellular organization within the 4D‐printed constructs.

To evaluate cytoskeletal organization as a marker of cell function, we examined filamentous actin (F‐actin) organization (Figure 6G,H and Figures S30–S33). Fluorescence imaging on day 7 revealed heterogeneous F‐actin organization, including circular patterns consistent with circumferential actin bundles surrounding spherical cell boundaries or radial/transverse arc‐like structures, irregular diffuse networks, and occasional aligned filaments—indicative of asynchronous, early‐stage cytoskeletal organization (Figure 6G and Figure S30) [45, 46, 47, 48, 49]. By day 14, cells developed prominent, well‐aligned F‐actin stress fibers spanning the cytoplasm, reflecting enhanced polarization and mechanotransduction (Figure 6G,H and Figure S31). Consistent with the coordinated alignment of elongated cells, actin stress fibers showed coherent orientation across adjacent cell populations, particularly in larger GelMA domains, supporting local cytoskeletal coordination (Figure 6G,H and Figure S31). This progression toward aligned actin architecture denotes coordinated cytoskeletal maturation across cells and a collective shift to more mechanically active cellular states. Nuclei also became elongated, suggesting increased cytoskeletal tension and effective force transmission through the actin cytoskeleton (Figures S31–S33) [50, 51]. In parallel, cell migration out of GelMA domains by day 14 supports a mechanically active and motile state (Figure 6F).

Together, these results demonstrate the cytocompatibility of our 4D bioprinting workflow and the functional cellular activity supported in the resulting 4D‐printed constructs. The HAMA–pNIPAm and PEGDA–pNIPAm systems (Figures S5 and S6) further support that the discrete material programming approach can, in principle, be extended to other cell‐compatible domain–matrix combinations. Its implementation requires an actuating matrix, a comparatively non‐actuating cell‐supportive domain phase with sufficient stiffness to locally constrain matrix actuation, and robust interfacial coupling at the domain–matrix interface.

## 3 Conclusion

Inspired by biological morphogenesis, we demonstrate cell‐compatible discrete 2D material programming for growth‐driven 3D shaping and morphogenesis‐inspired 4D bioprinting. By integrating programmable in‐plane growth with cell‐supportive microdomains, we enable the 4D bioprinting of living constructs with doubly curved morphologies—prevalent in living organisms but challenging to fabricate using conventional methods. The transformed constructs maintain high cell viability and support tissue‐relevant cellular behaviors. For many biological applications, cell‐laden constructs are transformed to the target geometry and then maintained at 37°C; in this collapsed pNIPAm state, the matrix is mechanically stiffer, supporting structural integrity over prolonged operation under physiological conditions. Design rules based on integration, transformation, and modular assembly of target metrics further broaden the programmable 3D shape space.

This study has limitations. Cells are initially confined to cell‐supportive domains and therefore do not uniformly populate the construct; more complete cellularization will require additional material and architectural strategies, although migration and matrix remodeling may extend cellular occupancy beyond patterned domains over time. In addition, we do not isolate curvature‐dependent cellular functional responses from baseline cellular behavior in GelMA using non‐morphing controls or constructs with prescribed curvatures; an important future study is to correlate programmed curvature and geometry with spatially resolved cellular functional responses.

The discrete programming concept is defined by the functional roles of the domain and matrix phases rather than by specific chemistries, providing a basis for extension to other cell‐compatible domain–matrix combinations and stimuli. Expanding the accessible shape space toward higher‐complexity architectures will require increasing the dynamic range of material programmability and leveraging advanced design strategies, including cone singularities and modular assembly of target‐metric modules within a sheet or across multiple programmed sheets [8]. From a biological perspective, integrating cell‐driven cues (e.g., cell‐generated forces or matrix degradation) with target‐metric programming is a promising route to time‐dependent shape evolution under physiological conditions. From an engineering perspective, in contrast to serial layer‐by‐layer deposition in conventional 3D and 4D bioprinting, the planar lithographic workflow enables parallel fabrication of multiple constructs with individualized programs, supporting scalable and customizable 4D bioprinting and offering opportunities for integration with established microfabrication workflows and device architectures. Cell‐compatible discrete 2D material programming provides a morphogenesis‐inspired biofabrication platform for hybrid living–synthetic systems, with potential applications in bioinspired soft robotics, engineered tissue constructs, and cell‐based devices.

## 4 Experimental Section

### 4.1 Preparation of Precursors and Bioinks

The precursor solution for pNIPAm hydrogels was prepared by dissolving *N*‐isopropylacrylamide (NIPAm; TCI America; 0.4 g), *N*,*N*′‐methylene bisacrylamide (1 mol% relative to NIPAm), poly(ethylene glycol) diacrylate (average molecular weight of ∼700 g mol^−1^; 0.125 mol% relative to NIPAm), and diphenyl(2,4,6‐trimethylbenzoyl)phosphine oxide (0.15 mol% relative to NIPAm) in 1 mL of a water/acetone mixture (1:3, v/v) [7, 8]. The precursor solution for GelMA hydrogels was prepared by dissolving GelMA (5 wt.%) and lithium phenyl‐2,4,6‐trimethylbenzoylphosphinate (LAP; Ambeed; 0.2 wt.%) in PBS (Corning) at 50°C. The GelMA precursor was equilibrated to 37°C prior to printing. For the cell‐encapsulated GelMA precursor (bioink), 3T3 fibroblasts were dispersed in the GelMA precursor solution at 2 × 10^6^ cells mL^−1^ and gently mixed at 37°C. GelMA was synthesized by reacting gelatin (type A, 300 g Bloom, porcine skin) with methacrylic anhydride following a published protocol [52]. The degree of methacryloyl modification (74.3%) was quantified by ^1^H NMR spectroscopy (Figure S34) [36, 53]. HAMA was synthesized by reacting hyaluronic acid (average molecular weight of ∼70–80 kDa; Biosynth) with methacrylic anhydride as described previously [36]. Unless otherwise noted, reagents were purchased from Sigma–Aldrich.

### 4.2 Cell Culture

NIH/3T3 fibroblasts (CRL‐1658, ATCC) were cultured in Dulbecco's modified Eagle medium (DMEM; Corning) supplemented with 10% fetal bovine serum (FBS; Corning) and 1% penicillin–streptomycin (Gibco) at 37°C and 5% CO_2_. This medium was used for all cell culture experiments. Prior to bioink preparation, cells cultured in flasks or dishes were detached, counted, and assessed for viability using a hemocytometer with trypan blue exclusion.

### 4.3 Discrete 2D Material Programming for 3D Shaping

The projection lithography cell was assembled by placing a 400 µm‐thick polydimethylsiloxane (PDMS) spacer on a PDMS substrate and covering the spacer with a 150 µm‐thick cover glass. Planar composite hydrogels programmed with Ω were fabricated by multimaterial digital light lithography [8]. After loading the pNIPAm precursor into the lithography cell, a patterned pNIPAm matrix was formed by photocrosslinking the precursor for 8 s using spatially controlled illumination from a digital light processing projector (Vivitek D912HD; measured intensity, ∼32.5 mW cm^−2^ at 405 nm and ∼0.4 mW cm^−2^ at 365 nm) during the first printing step. After the first printing, the crosslinked pNIPAm matrix remained attached to the cover glass. The cover glass was lifted, with one side fixed using a tape hinge (Scotch tape, 3m) to align the first‐print pattern with the second printing step. The patterned pNIPAm matrix and the lithography cell were washed twice with isopropanol and once with ethanol. After the pNIPAm matrix was dried, the GelMA precursor was loaded into the cell, which was closed by lowering the cover glass. GelMA domains were patterned by photocrosslinking the GelMA precursor for 12 s using spatially controlled illumination during the second printing step. The resulting composite hydrogels were washed three times with PBS, detached from the cover glass, and incubated in PBS at 37°C to transform into the programmed 3D shapes. The transformed constructs were imaged after ∼16 h of incubation, unless otherwise stated. For the HAMA–pNIPAm system, HAMA domains were patterned during the second printing step by photocrosslinking either a HAMA precursor containing 1 wt.% HAMA for 16 s or a HAMA precursor containing 1.25 wt.% HAMA for 28 s. For the PEGDA–pNIPAm system, PEGDA domains were patterned during the second printing step by photocrosslinking a PEGDA precursor containing 5 wt.% PEGDA (average molecular weight of ∼700 g mol^−1^) for 16 s. The first and second printing patterns were generated from Ω in 3ds Max (Autodesk). To transform constructs from the shrunken state at 37°C to the swollen state at room temperature, the PBS was cooled to room temperature.

### 4.4 Gaussian Curvature Measurement

Morphed constructs were imaged from two orthogonal side views (0° and 90°) to obtain perpendicular cross‐sectional profiles through the central region of each construct, corresponding to the two principal curvature directions. In each view, the boundary profile was extracted by edge detection and fit to a circular arc to determine the radius of curvature *ρ*. Fits were performed over the central region to minimize edge effects. The principal curvatures were computed as *k*_1_ = 1/*ρ*_1_ and *k*_2_ = 1/*ρ*_2_, and the Gaussian curvature as *K* = *k*_1_ *k*_2_.

### 4.5 4D Bioprinting

For bioprinting, the cell‐encapsulated GelMA precursor (bioink) was used for the second printing step. After printing, constructs were washed three times with PBS and incubated in complete cell culture medium at 37°C and 5% CO_2_ to enable shape transformation and cell culture within the printed constructs. After 2 h, the medium was replaced with fresh medium and refreshed every 2 days thereafter. Non‐morphing planar disk controls (multimaterial printing without shape transformation) were fabricated by printing a cell‐laden circular GelMA domain (4.5 mm in diameter) within a printed pNIPAm disk (12 mm in diameter). Non‐morphing planar GelMA‐only disk controls (single‐material printing without a pNIPAm matrix) were fabricated by printing cell‐laden GelMA disks (4 mm in diameter).

### 4.6 Measurement of Areal Swelling and Shrinking Ratios

Composite hydrogel disks (12 mm diameter) containing GelMA domains with a uniform diameter within each disk were fabricated using the 4D‐printing procedure described above (Figure 1C and Figure S1). The GelMA domain diameter was varied to obtain area fractions *ϕ*_G_ spanning 0 to 0.5. The disk areas in the shrunken (*A*_37_) and swollen (*A*_25_) states were quantified from top‐view images acquired at 37°C and 25°C, respectively. The areal shrinkage and swelling ratios were defined as *A*_37_/*A*_0_ and *A*_25_/*A*_0_, respectively, where *A*_0_ is the area of the as‐printed disks. Calibration curves (Figure 1D,E) were generated by plotting *A*_37_/*A*_0_ and *A*_25_/*A*_0_ as functions of *ϕ*_G_, respectively.

### 4.7 Estimation of GelMA Elastic Modulus by Rheology

The shear storage modulus (*G*′) and loss modulus (*G*″) of photocrosslinked GelMA (*n* = 3 independent samples) were measured using a rheometer (DHR‐2, TA Instruments) with an 8‐mm‐diameter parallel‐plate geometry. Frequency sweeps (0.1–10 Hz) were performed within the linear viscoelastic region (strain = 0.5%), and *G*′ and *G*″ values were evaluated at 1 Hz. The elastic modulus *E* (1.8 kPa) was estimated using *E* = 2*G*(1 + *ν*) with *ν* = 0.5 (Poisson's ratio), where *G* = (*G*′^2^ + *G*″^2^)^1/2^ ≈ *G*′.

### 4.8 Cell Viability Assay

Cell viability in 4D‐bioprinted constructs was quantified using three‐channel fluorescence staining: live cells (green) and dead cells (red) were stained with a Live/Dead viability/cytotoxicity kit (Biotium), and total nuclei (blue) were counterstained with Hoechst 33342 (Invitrogen), following the manufacturers’ instructions. Samples were imaged using a fluorescence microscope (Axio Observer Z1, Zeiss). In conditions where individual live cells could not be reliably quantified (e.g., multicellular assemblies), viability was calculated from dead‐cell and total‐nuclei counts: viability (%) = (1 – *N*_dead_/*N*_total_) × 100.

### 4.9 Metabolic Activity Assay

Cellular metabolic activity in 4D‐bioprinted constructs was quantified using an alamarBlue cell proliferation assay (Bio‐Rad) following the manufacturer's instructions. Fluorescence intensity was measured with a microplate reader (Varioskan LUX, Thermo Scientific) to assess cellular metabolic activity.

### 4.10 F‐Actin Staining and Imaging

F‐actin was visualized by phalloidin staining (Cytoskeleton) according to the manufacturer's instructions. Samples were fixed in 4% formalin, permeabilized with 0.1% Triton X‐100 in PBS, and stained with fluorescent phalloidin. Nuclei were counterstained with Hoechst 33342. Samples were imaged using a fluorescence microscope.

### 4.11 Statistical Analysis

Quantitative data are reported as mean ± s.d. All *n* values denote independent samples (independent constructs). Statistical comparisons were performed using one‐ or two‐way analysis of variance (ANOVA) followed by Tukey's multiple‐comparisons test. Statistical significance was defined as *p* < 0.05.

## Author Contributions

**Fereshteh Family**: investigation, writing original draft, methodology, validation, visualization, writing – review and editing, data curation, formal analysis. **Aneela Davuluri**: investigation, writing original draft, methodology, validation, visualization, writing – review and editing, data curation, formal analysis. **Athulya Martin**: methodology, validation, visualization, writing – review and editing. **Kyungsuk Yum**: conceptualization, writing – original draft, funding acquisition, methodology, visualization, validation, writing – review and editing, project administration, formal analysis, supervision, resources.

## Funding

This work was supported by the National Science Foundation (DMR‐1848511, CMMI‐2221603, DMR‐242516)

## Conflicts of Interest

The authors declare no conflicts of interest.

## Supporting information




**Supporting File 1**: advs77228‐sup‐0001‐SuppMat.pdf.

**Supporting File 2**: advs77228‐sup‐0002‐Movie1.mp4.

**Supporting File 3**: advs77228‐sup‐0003‐Movie2.mp4.

**Supporting File 4**: advs77228‐sup‐0004‐Movie3.mp4.

**Supporting File 5**: advs77228‐sup‐0005‐Movie4.mp4.

**Supporting File 6**: advs77228‐sup‐0006‐Movie5.mp4.

**Supporting File 7**: advs77228‐sup‐0007‐Movie6.mp4.

**Supporting File 8**: advs77228‐sup‐0008‐Movie7.mp4.

## Data Availability

The data that support the findings of this study are available from the corresponding author upon reasonable request.
