# Supplementary material for: Discrete 2D Material Programming for 3D Shaping and Morphogenesis‐Inspired 4D Bioprinting
Source: Adv Sci (Weinh). 2026 Aug 21;13(62):e77228. doi: 10.1002/advs.77228 (PMC13496281; doi:10.1002/advs.77228)
Supplement: Supplementary file 1 [file ADVS-13-e77228-s007.pdf]

Supporting Information

Discrete 2D Material Programming for 3D Shaping and Morphogenesis-Inspired 4D Bioprinting

*Fereshteh Family, Aneela Davuluri, Athulya Martin, and Kyungsuk Yum**

Supporting Text

Shape-guiding modules

Metrics associated with complex morphing patterns or assemblies of multiple modules can admit multiple isometric embeddings, often exhibiting reflection-related degeneracy, leading to distinct global shapes under the same Ω [1-3]. For example, in modular assemblies, each module may morph in different directions (e.g., upward or downward) or adopt different orientations relative to neighboring modules, unless constraints exist [2, 3]. In our 4D-printing method, a slight through-thickness gradient in hydrogel shrinkage provides an inherent symmetry-breaking constraint that reduces the number of accessible embeddings and enables robust realization of the intended shapes across diverse geometries (Figures 2 and 3) [2, 3]. However, this constraint does not allow independent control of module directions.

To actively coordinate shape selection in assemblies of modular target metrics, we adopted the concept of shape-guiding modules [2, 3]. A shape-guiding module is a localized metric feature with small $|K|$ that couples the morphing directions of adjacent regions while minimally perturbing the global target shape. In the four-spherical-cap assemblies (Figure 4B–E), Ω with shape-guiding modules produced constructs in which each cap domain deformed in its programmed upward or downward direction (Figure 4C–E), whereas an unguided assembly yielded a construct in which all cap domains deformed in a single direction (Figure 4B). In these designs, modular components sharing edges aligned with the long (parallel) axis of a shape-guiding module with $K < 0$ are biased to deform in the same direction, whereas components coupled across the orthogonal (perpendicular) axis are biased to deform in opposite directions (Figure 4C–E). A shape-guiding module with $K > 0$ likewise synchronizes the morphing directions of adjacent modular components coupled along its parallel edges (Figures 4F and 5E,F).

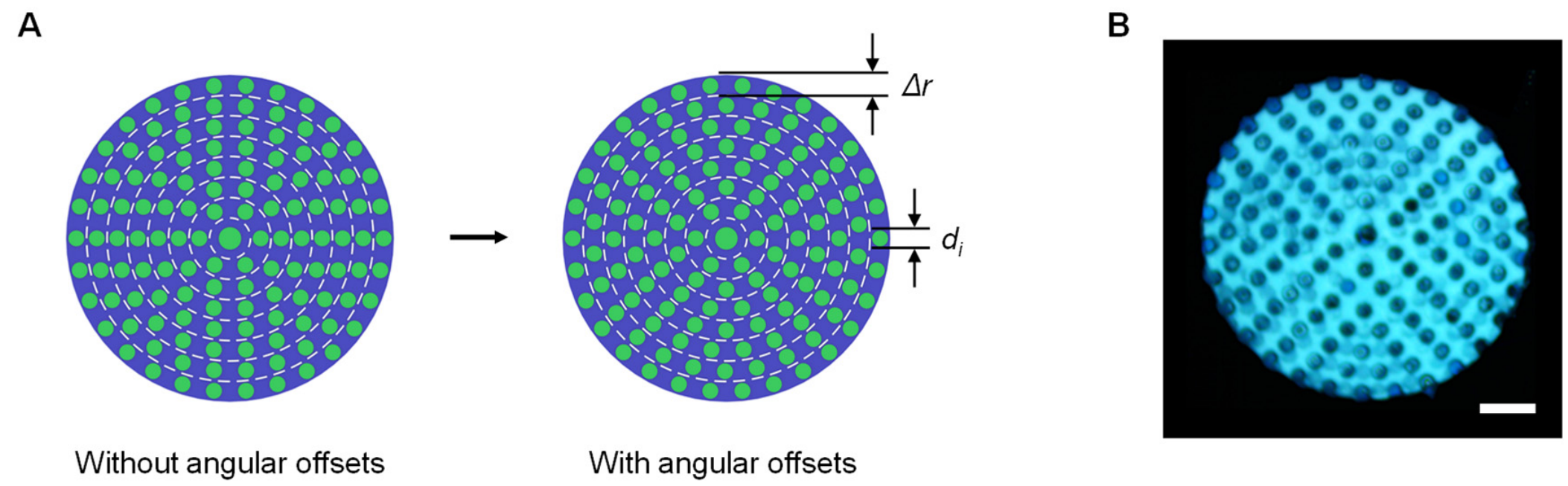

Figure S1. Material programming platform. (A) Axisymmetric material programming platform comprising a central disk of radius Δr containing a single GelMA circle of diameter d_0, surrounded by annuli of radial thickness Δr, each containing n_i GelMA circles of diameter d_i, distributed uniformly along θ. To promote uniform spatial distribution, the angular positions of GelMA circles are alternated between adjacent annuli by applying an angular offset of $2\pi/n_\mathrm{i}$ to every other annulus. For example, the GelMA circles in the first annulus are aligned with no angular offset, whereas those in the second annulus with $n_2 = 10$ are offset by 0.2π. Left and right schematics show the platform without and with angular offsets, respectively ($\phi_\mathrm{G} = 0.3$). (B) Representative image of a composite hydrogel disk with uniform d_i ($\phi_\mathrm{G} = 0.3$). Scale bar, 2 mm.

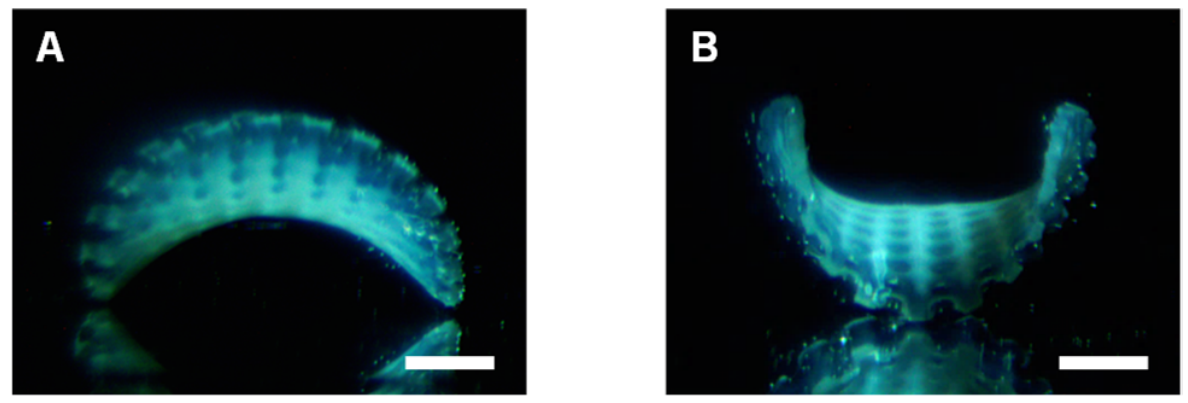

Figure S2. Orthogonal side views of a saddle structure. (A) Side view of the saddle structure in Figure 2C along one principal direction, highlighting one principal curvature. (B) Side view of the same saddle structure along the orthogonal principal direction, highlighting the other principal curvature. Scale bars, 2 mm.

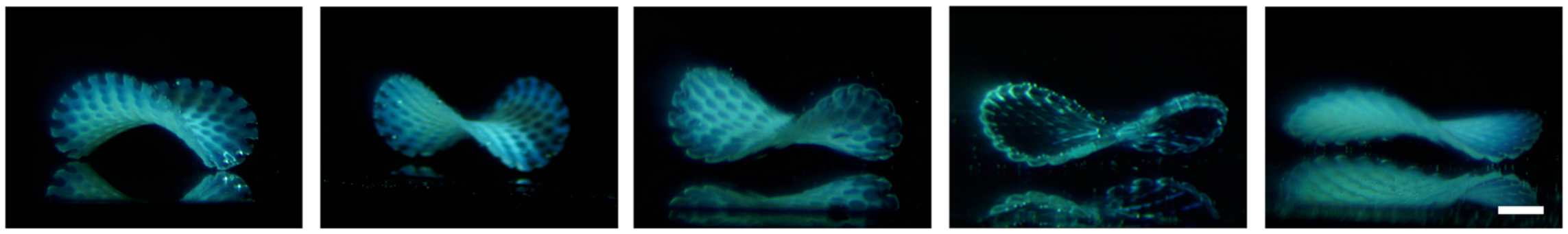

Figure S3. Saddle structures programmed with varying *K*. Representative saddle structures programmed with target Gaussian curvature K_{prog} of −0.090, −0.071, −0.052, −0.033, and −0.016 mm^{-2} (from left to right). Scale bar, 2 mm.

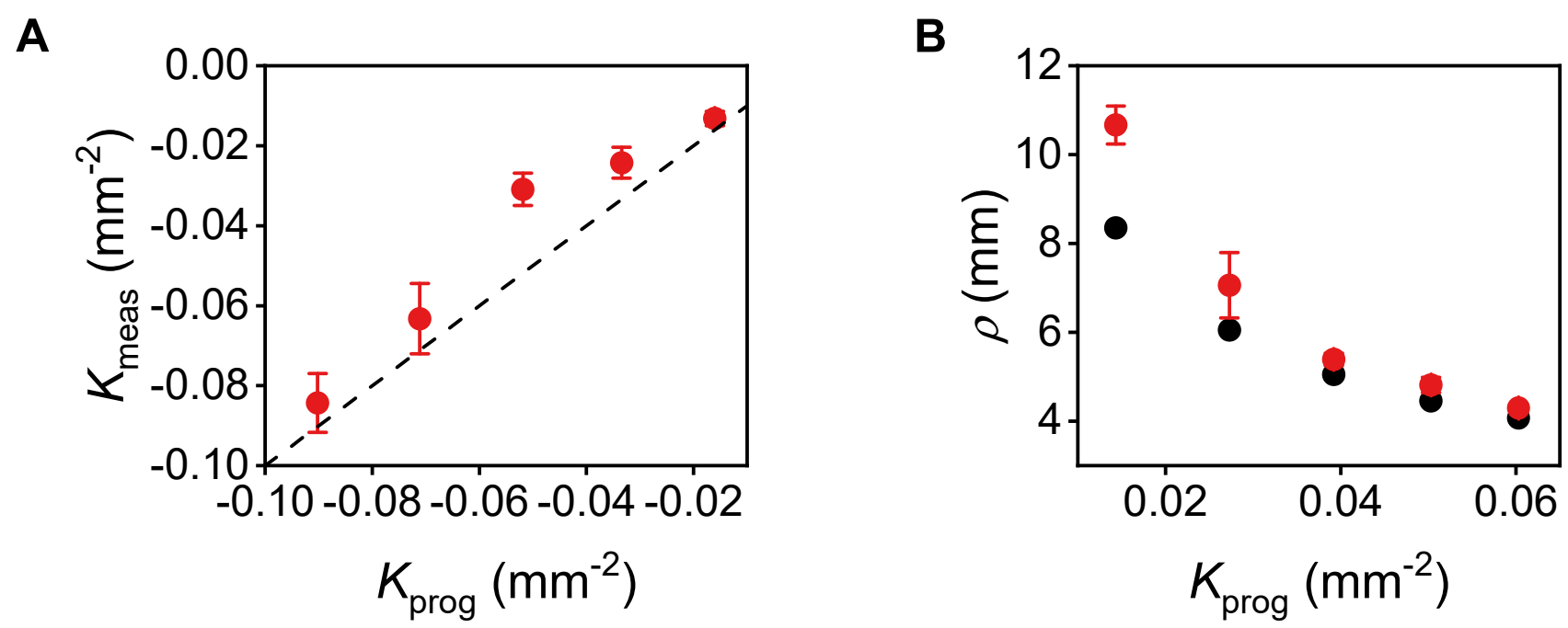

Figure S4. Curvature validation for saddle and spherical-cap structures. (A) Measured Gaussian curvature K_{meas} of saddle structures programmed with target Gaussian curvature K_{prog} = −0.090, −0.071, −0.052, −0.033, and −0.016 mm^{-2} (red circles). The dashed line indicates the theoretical K. (B) Measured (red circles) and theoretical (black circles) radius of curvature ρ of spherical-cap structures programmed with target K_{prog} = 0.014, 0.027, 0.039, 0.050, and 0.060 mm^{-2}. Data are mean ± s.d (n = 3 independent structures).

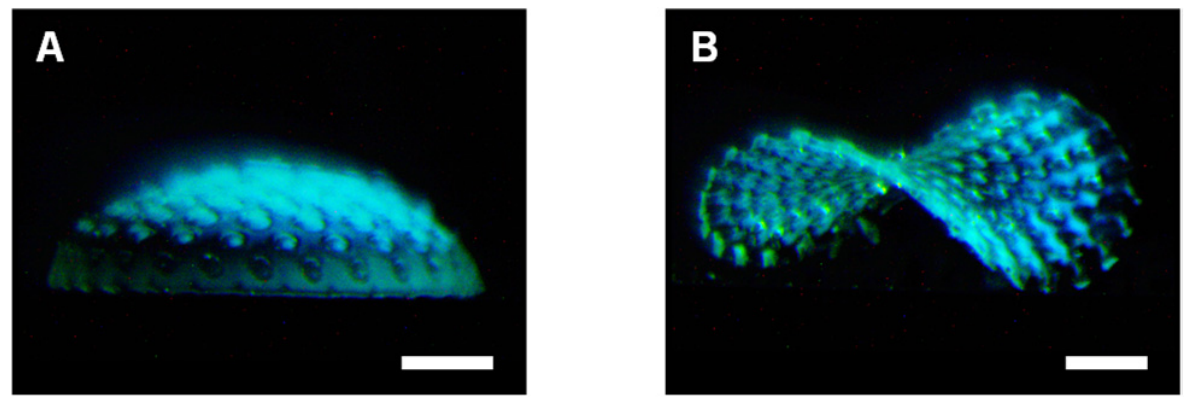

Figure S5. Growth-driven 3D shaping using HAMA domains within pNIPAm matrices.

(A) Formation of a spherical-cap structure with constant positive Gaussian curvature ($K > 0$).

(B) Formation of a saddle structure with constant negative Gaussian curvature ($K < 0$).

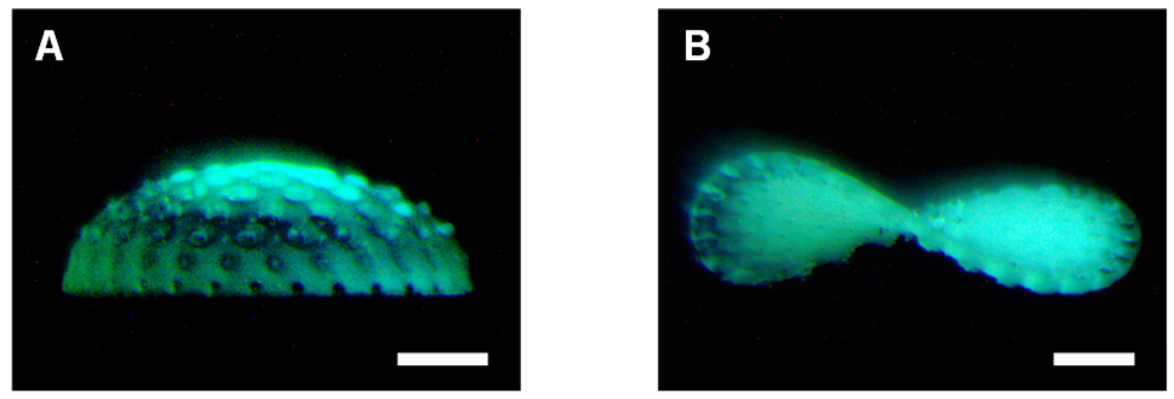

Figure S6. Growth-driven 3D shaping using PEGDA domains within pNIPAm matrices.

(A) Formation of a spherical-cap structure with constant positive Gaussian curvature ($K > 0$).

(B) Formation of a saddle structure with constant negative Gaussian curvature ($K < 0$).

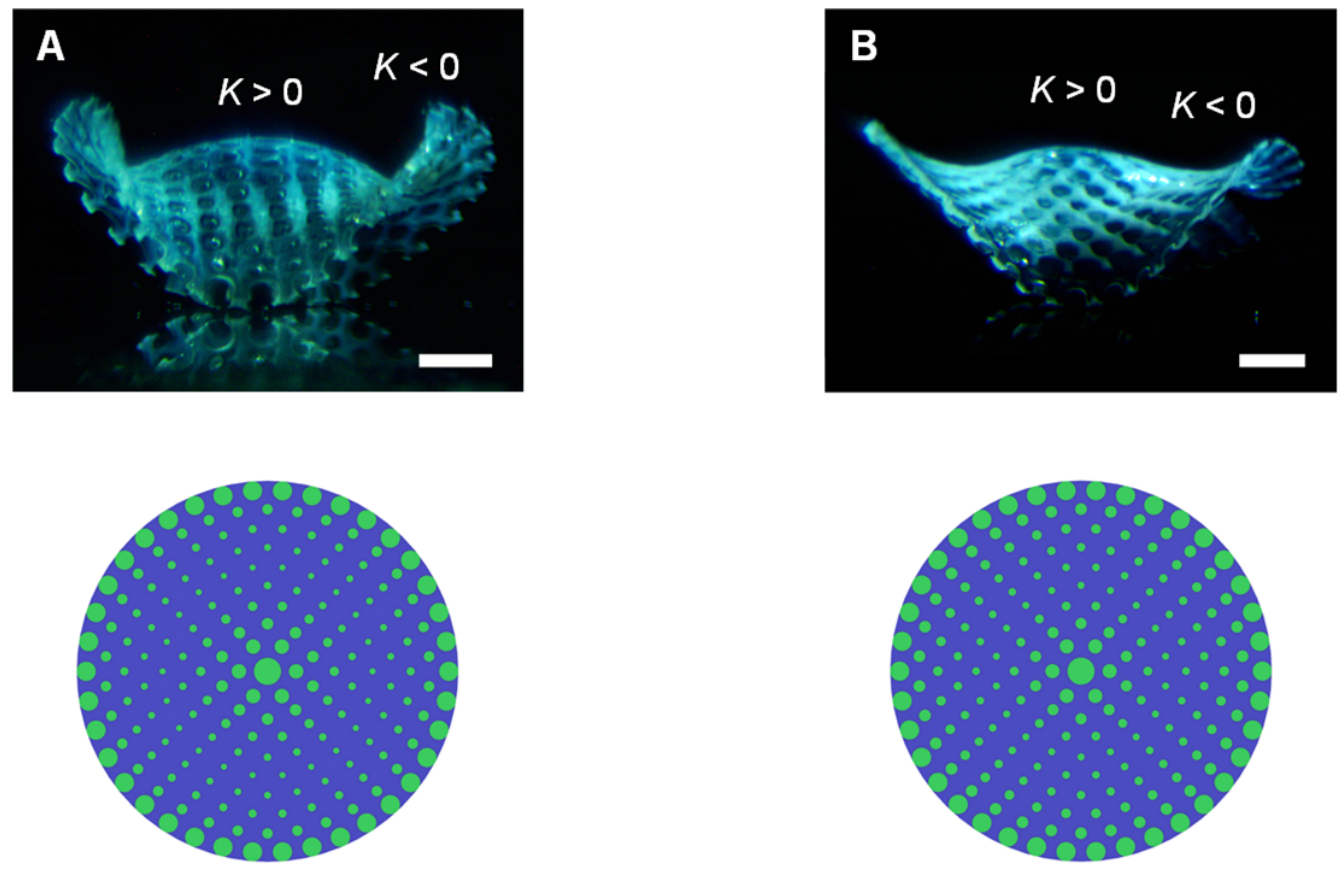

Figure S7. Hybrid constructs generated by radial integration of target metrics. (A) Hybrid construct exhibiting $K > 0$ in the central region and $K < 0$ in the periphery, generated by radial integration of Ω_1 for a spherical cap (i = 0–6; Ω_r = 2.24) and Ω_2 for a saddle (i = 6–10; Ω_r = 2.24), with the corresponding printing pattern used for material programming. (B) Hybrid construct exhibiting $K > 0$ in the central region and $K < 0$ in the periphery, generated by radial integration of Ω_1 for a spherical cap (i = 0–5; Ω_r = 1.90) and Ω_2 for a saddle (i = 5–10; Ω_r = 2.07), with the corresponding printing pattern used for material programming. Scale bars, 2 mm.

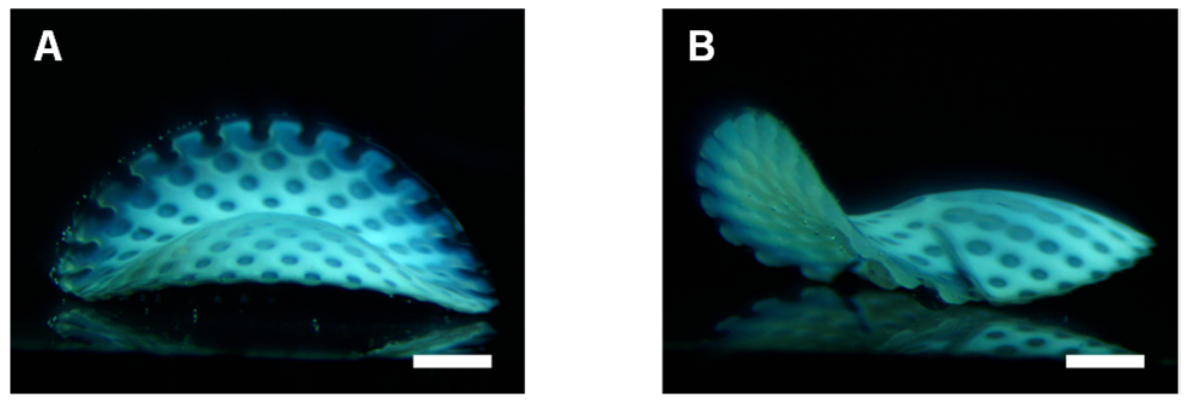

Figure S8. Hybrid construct generated by azimuthal integration of target metrics. (A) Side view of the construct in Figure 3C, viewed toward the midpoint of the $K > 0$ domain ($\theta = \pi/2$). (B) Side view of the same construct, viewed toward the $K < 0$ domain near $\theta \approx 3\pi/2$. Scale bars, 2 mm.

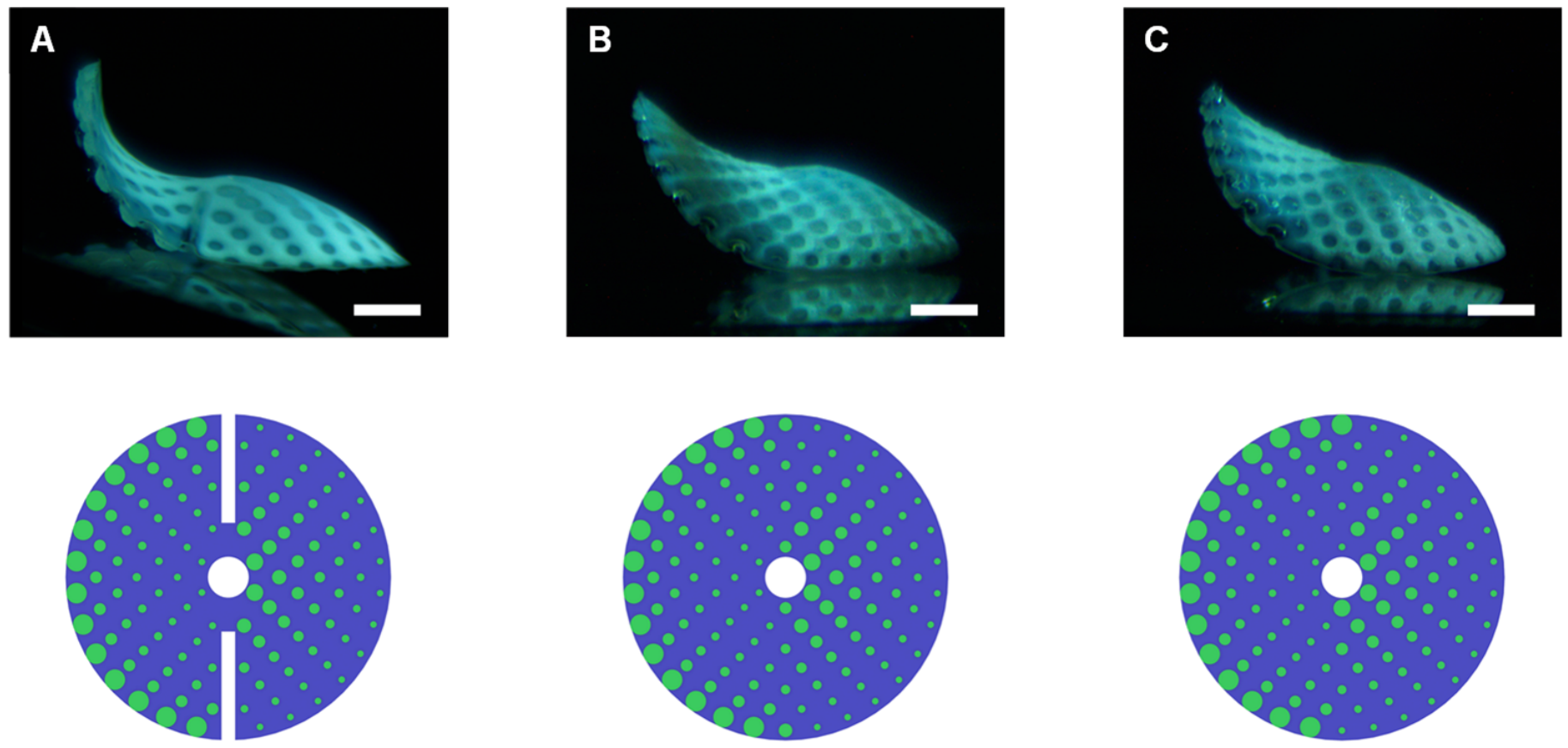

Figure S9. Hybrid constructs generated by azimuthal integration of target metrics with and without interfacial cuts and linkers. (A) Side view of the hybrid construct with interfacial cuts shown in Figure 3C, viewed toward one of the two $K > 0$ and $K < 0$ interfaces ($\theta = 0$), and the corresponding printing pattern used for material programming. (B) Side view of the hybrid construct with interfacial linkers, viewed toward the $K > 0$ and $K < 0$ interface ($\theta = 0$), and the corresponding printing pattern used for material programming. (C) Side view of the hybrid construct without cuts or linkers, viewed toward the $K > 0$ and $K < 0$ interface ($\theta = 0$), and the corresponding printing pattern used for material programming. All constructs were generated by azimuthal integration of the same target metrics (Ω_1 and Ω_2). Experimentally measured principal curvatures are compared with theoretical values in Table S1. Scale bars, 2 mm.

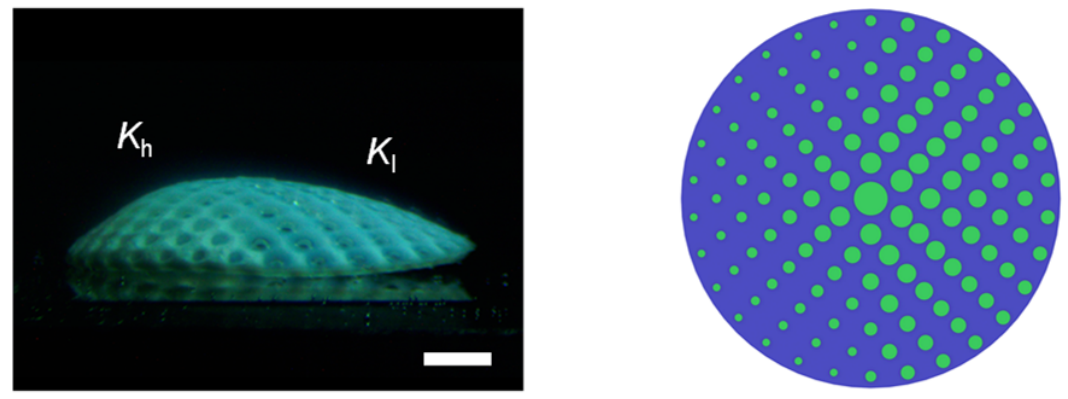

Figure S10. Hybrid construct generated by azimuthal integration of two spherical-cap target metrics with different Gaussian curvatures. Hybrid construct formed by azimuthal integration of Ω_1 for a spherical cap with lower K (K_l; $0 < \theta < \pi$) and Ω_2 for a spherical cap with higher K (K_h; $\pi < \theta < 2\pi$) using interfacial linkers (left), with the corresponding printing pattern used for material programming (right). Scale bar, 2 mm.

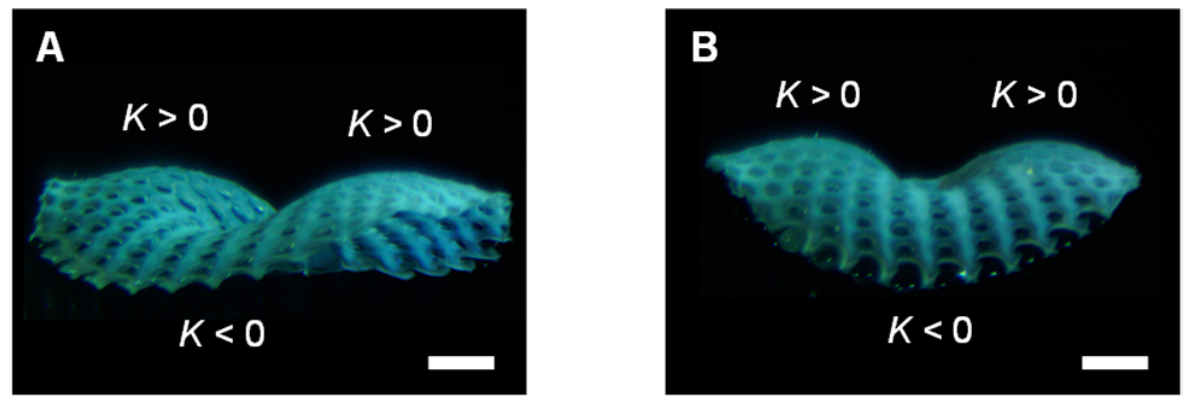

Figure S11. Four-domain construct exhibiting alternating *K* > 0 and *K* < 0 regions. (A) Additional view of the construct shown in Figure 3D. (B) Side view of the same construct. Scale bar, 2 mm.

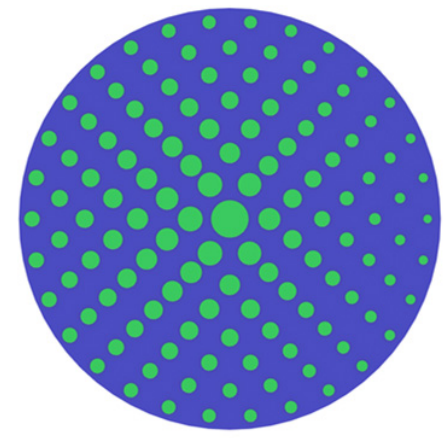

Figure S12. Printing pattern used for the construct with smoothly varying *K* along *θ*. The printing pattern was used to program the construct shown in Figure 3H.

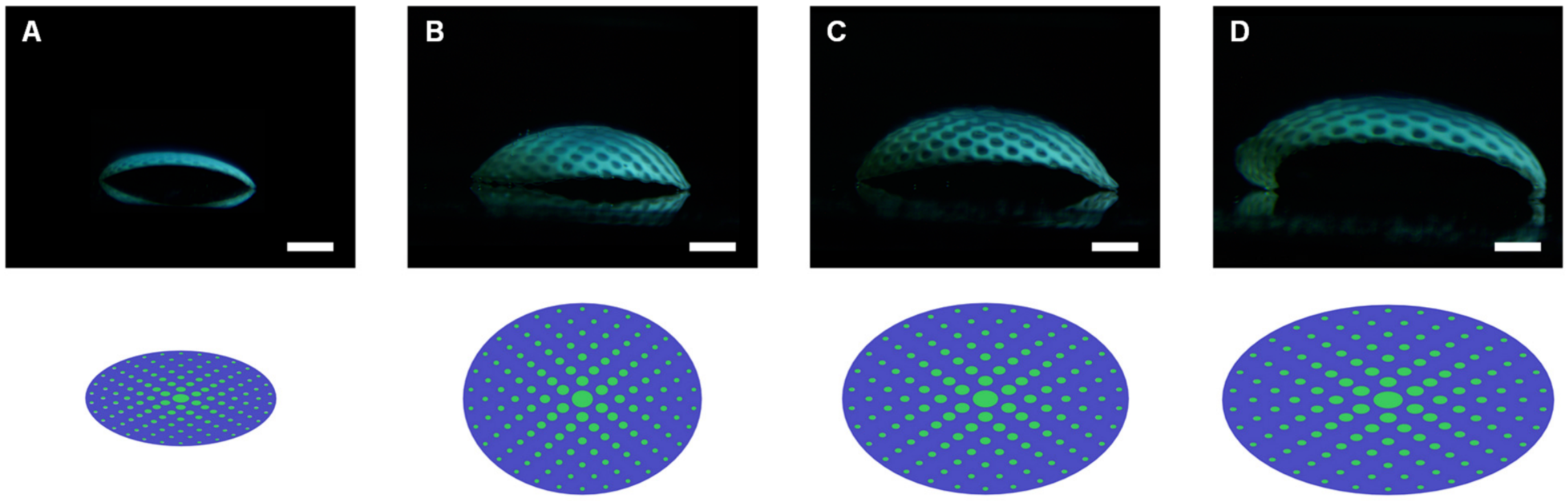

Figure S13. Elongated spherical-cap constructs. Elongated spherical-cap constructs generated using printing patterns obtained by elliptically deforming the spherical-cap pattern (Figure 2B), with semi-major and semi-minor axes r_1 and r_2, respectively (construct, top; printing pattern, bottom). (A) Aspect ratio (AR = r_1/r_2) = 2: $r_1 = r$, $r_2 = 0.5r$, where r is a constant. (B) AR = 1.25: $r_1 = 1.25r$, $r_2 = r$. (C) AR = 1.5: $r_1 = 1.5r$, $r_2 = r$. (D) AR = 1.75: $r_1 = 1.75r$, $r_2 = r$. Scale bars, 3 mm.

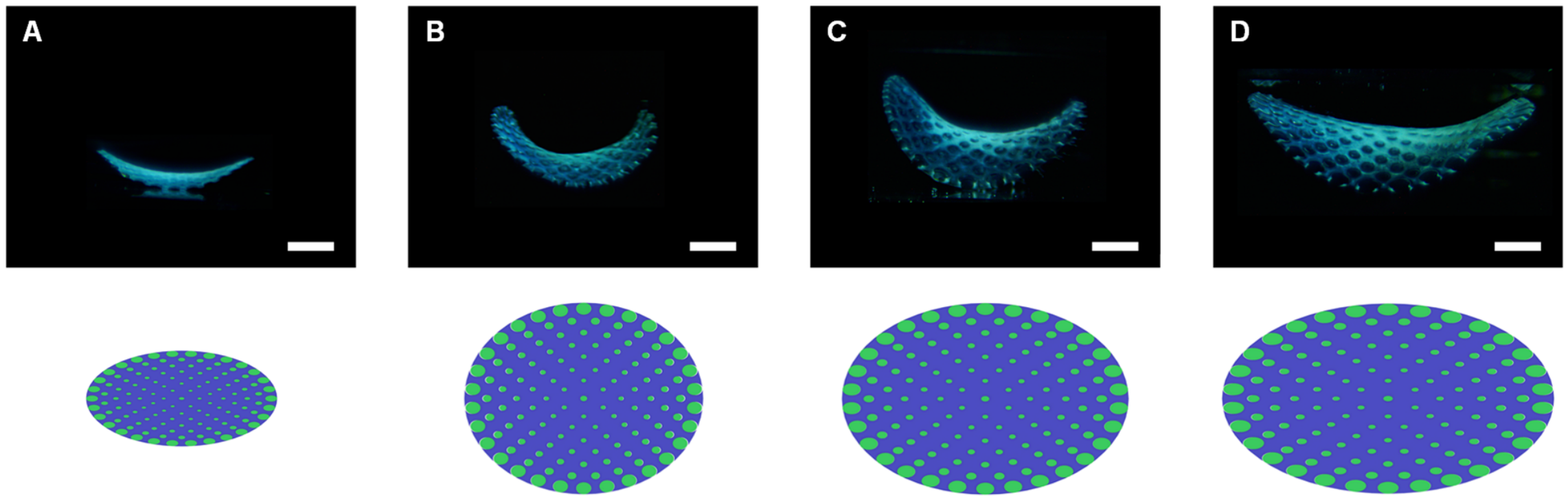

Figure S14. Elongated saddle constructs. Elongated saddle constructs generated using printing patterns obtained by elliptically deforming the saddle pattern (Figure 2D), with semi-major and semi-minor axes r_1 and r_2, respectively (construct, top; printing pattern, bottom). (A) Aspect ratio (AR = r_1/r_2) = 2: $r_1 = r$, $r_2 = 0.5r$, where r is a constant. (B) AR = 1.25: $r_1 = 1.25r$, $r_2 = r$. (C) AR = 1.5: $r_1 = 1.5r$, $r_2 = r$. (D) AR = 1.75: $r_1 = 1.75r$, $r_2 = r$. Scale bars, 3 mm.

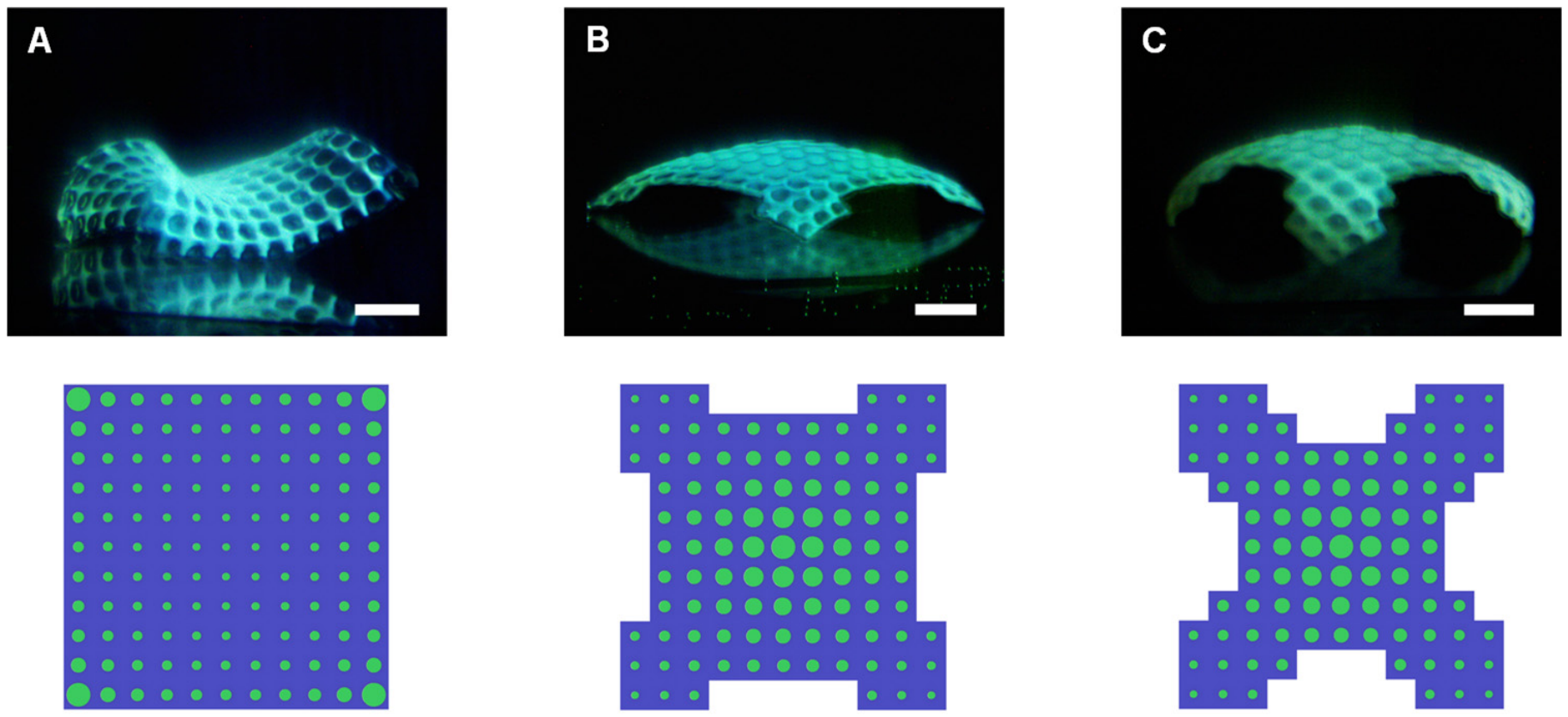

Figure S15. Constructs programmed with Ω(*x*, *y*). Constructs with programmable boundary geometries (top) generated using Ω(*x*, *y*) and the corresponding printing patterns used for material programming (bottom). (A) Saddle-like construct with a square boundary. (B) $K > 0$ construct with a square boundary and protruding corners. c, Construct comprising a $K > 0$ central body with four leg-like extensions. Scale bars, 2 mm.

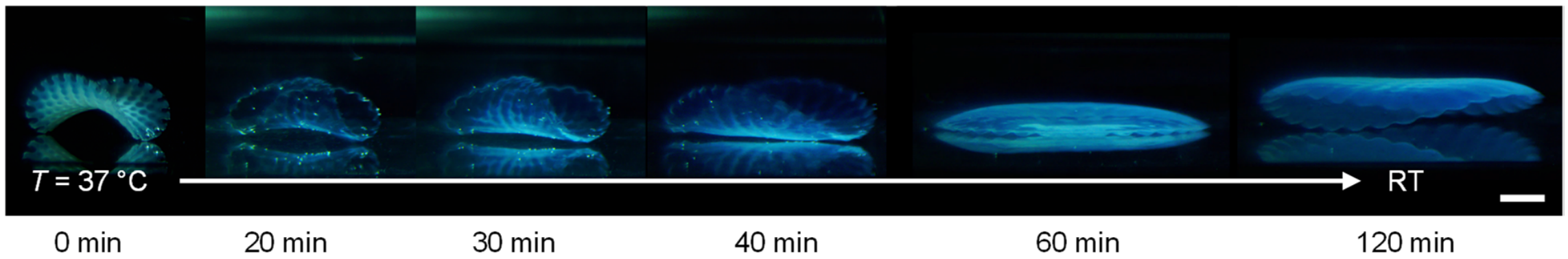

Figure S16. Shape evolution of a saddle structure during cooling. The construct underwent time-dependent morphing, progressing through a series of intermediate morphologies as the temperature decreased from 37 °C to room temperature (RT). Scale bar, 3 mm.

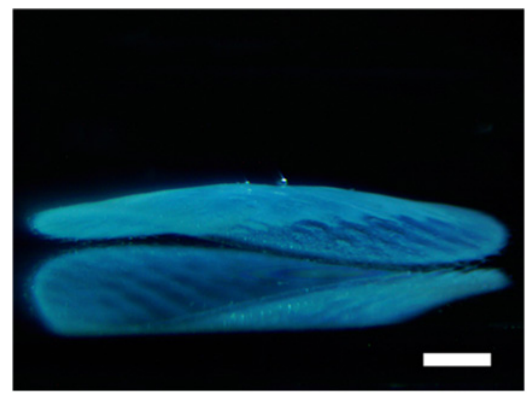

Figure S17. Spherical-cap construct 24 h after cooling. Configuration of the spherical-cap structure in Figure 5A after 24 h at room temperature. Scale bar, 3 mm.

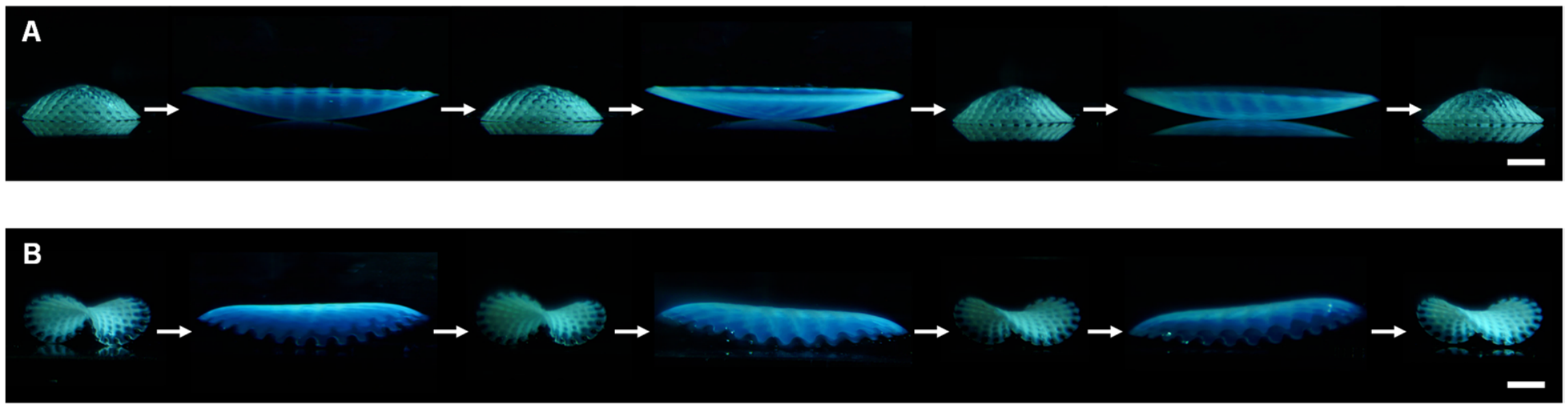

Figure S18. Reversible shape transformations. The shape-morphed constructs exhibited repeatable shape transformations over multiple swelling–shrinking cycles. (A) Reversible shape transformations of a spherical-cap structure. (B) Reversible shape transformations of a saddle structure. Scale bar, 3 mm.

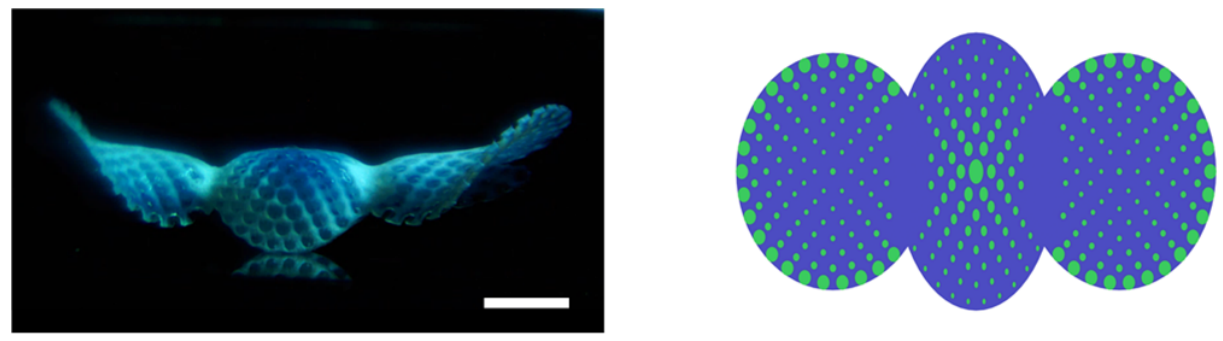

Figure S19. Stingray-inspired construct. Stingray-inspired construct used in Figure 5E,F (left) and corresponding printing pattern used for material programming (right). Scale bar, 3 mm.

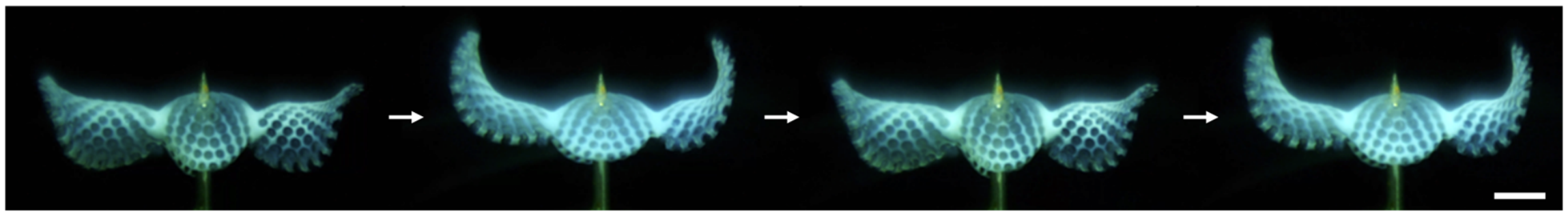

Figure S20. Oscillatory flapping motion of a stingray-inspired construct. The construct exhibited repeatable flapping under cyclic temperature modulation (28.5–34 °C). Scale bar, 3 mm.

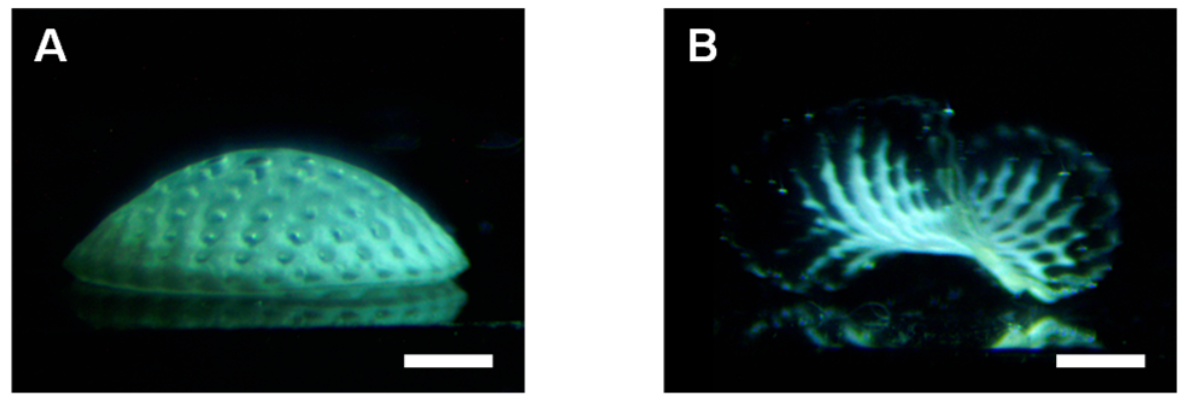

Figure S21. 4D-bioprinted constructs. (A) Representative image of a spherical-cap construct with cell-encapsulated GelMA domains on day 15 of culture. (B) Representative image of a saddle construct with cell-encapsulated GelMA domains on day 15 of culture. Scale bars, 2 mm.

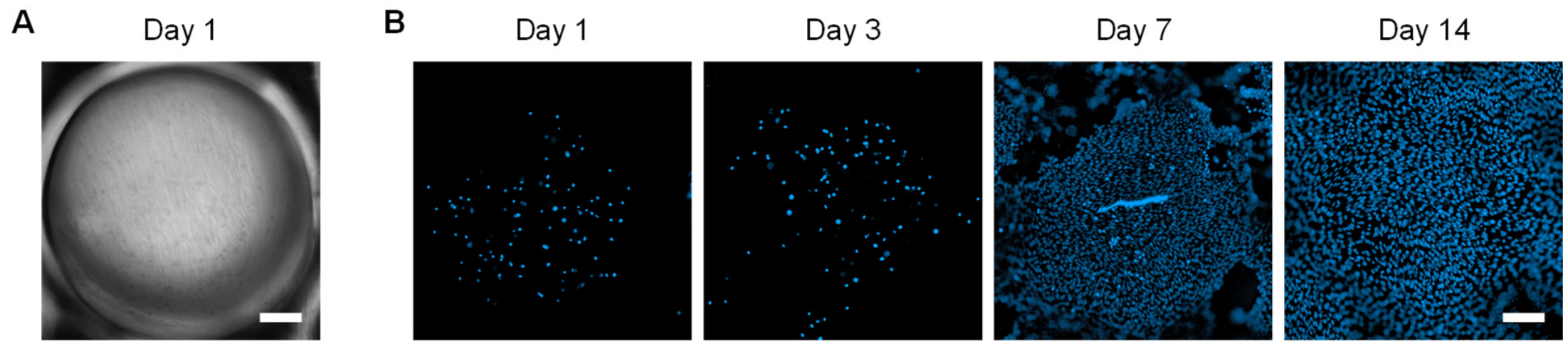

Figure S22. Representative images of GelMA domains in spherical-cap constructs. (A) Bright-field image of a GelMA domain on day 1, corresponding to the day-1 fluorescence image in Figure 6A. (B) Fluorescence images of nuclei (Hoechst 33342, blue) in and around GelMA domains ($\phi_G = 0.5$) of spherical-cap constructs on days 1, 3, 7, and 14 (different constructs), corresponding to the fluorescence images shown in Figure 6A. Scale bars, 200 μm.

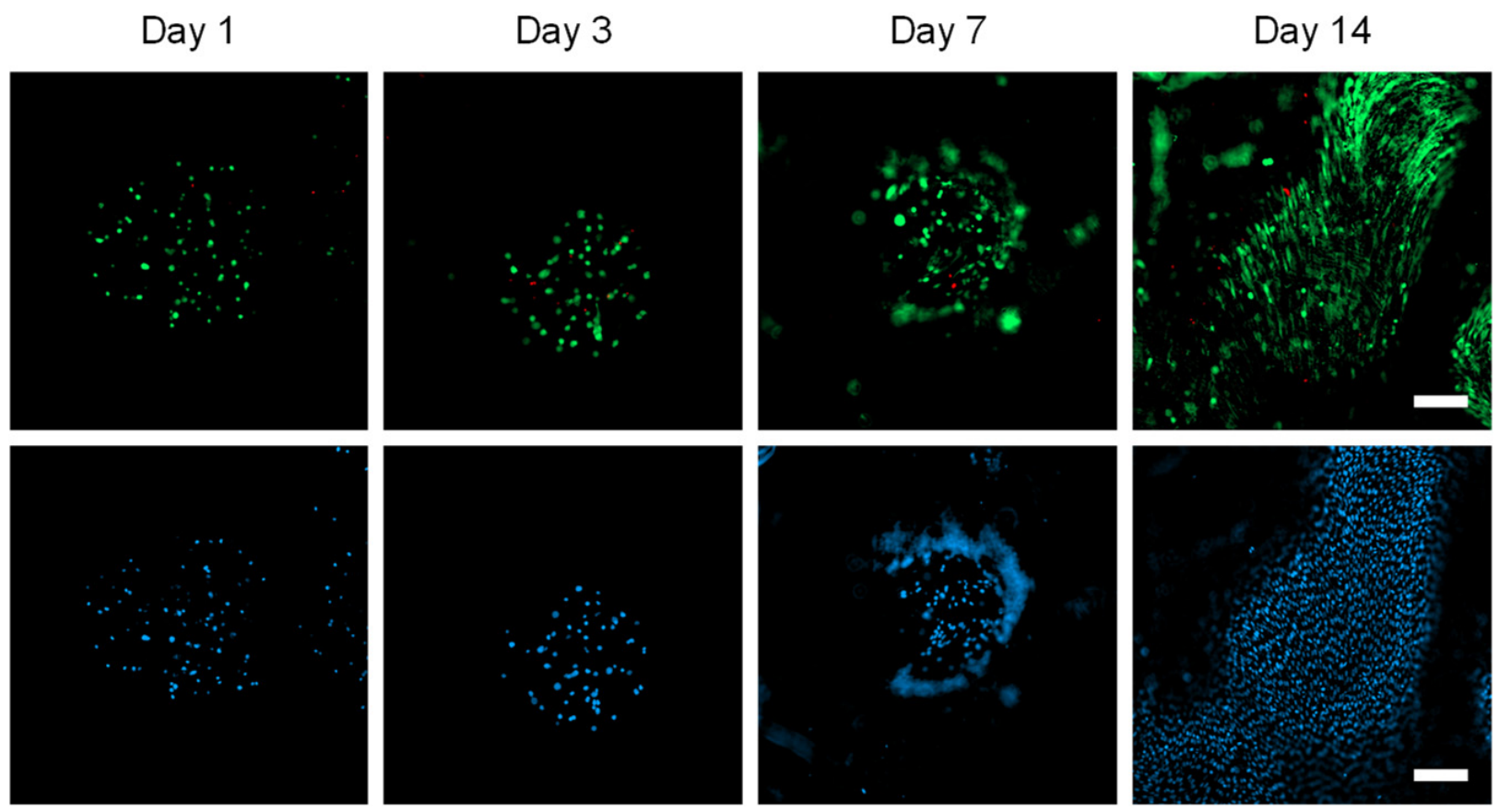

Figure S23. Representative fluorescence images of medium-sized GelMA domains in spherical-cap constructs. Representative images of fibroblasts in and around medium-sized GelMA domains ($\phi_G = 0.34$) of spherical-cap constructs on days 1, 3, 7, and 14 (different constructs). Live/Dead staining is shown in the top row (live, green; dead, red), and nuclei (Hoechst 33342, blue) are shown in the bottom row. Scale bars, 200 μm.

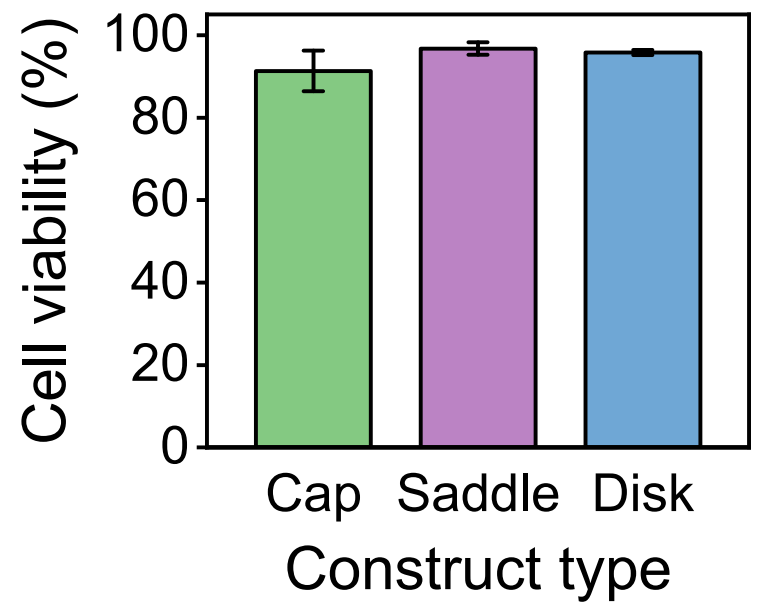

Figure S24. Cell viability on day 14 in different construct types. Viability on day 14 in spherical-cap constructs, saddle constructs, and non-morphing planar disk controls comprising a cell-laden GelMA domain embedded in a pNIPAm matrix. No statistically significant differences were detected among construct types (one-way ANOVA; $P = 0.1395$; $n = 4$ (spherical-cap), 3 (saddle), and 3 (disk) independent constructs).

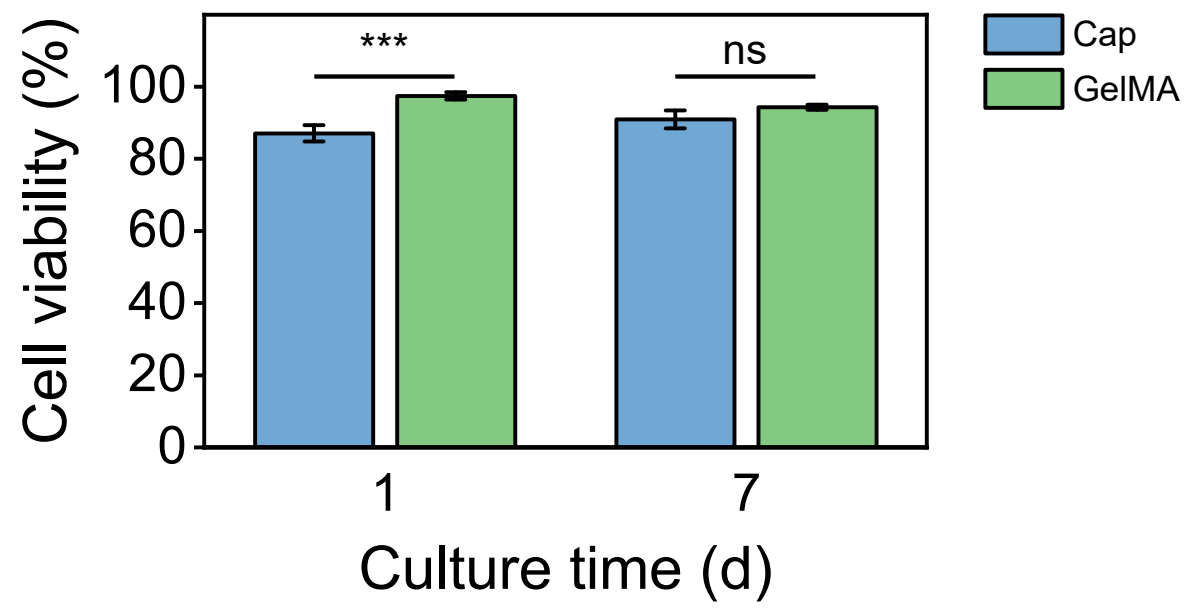

Figure S25. Viability in spherical-cap constructs versus non-morphing planar GelMA-only disk controls. Viability on days 1 and 7. Statistics: two-way ANOVA with Tukey's multiple-comparisons test; n = 4 (spherical-cap) and 3 (disk) independent constructs at each time point; ***P = 0.0009; ns, not significant.

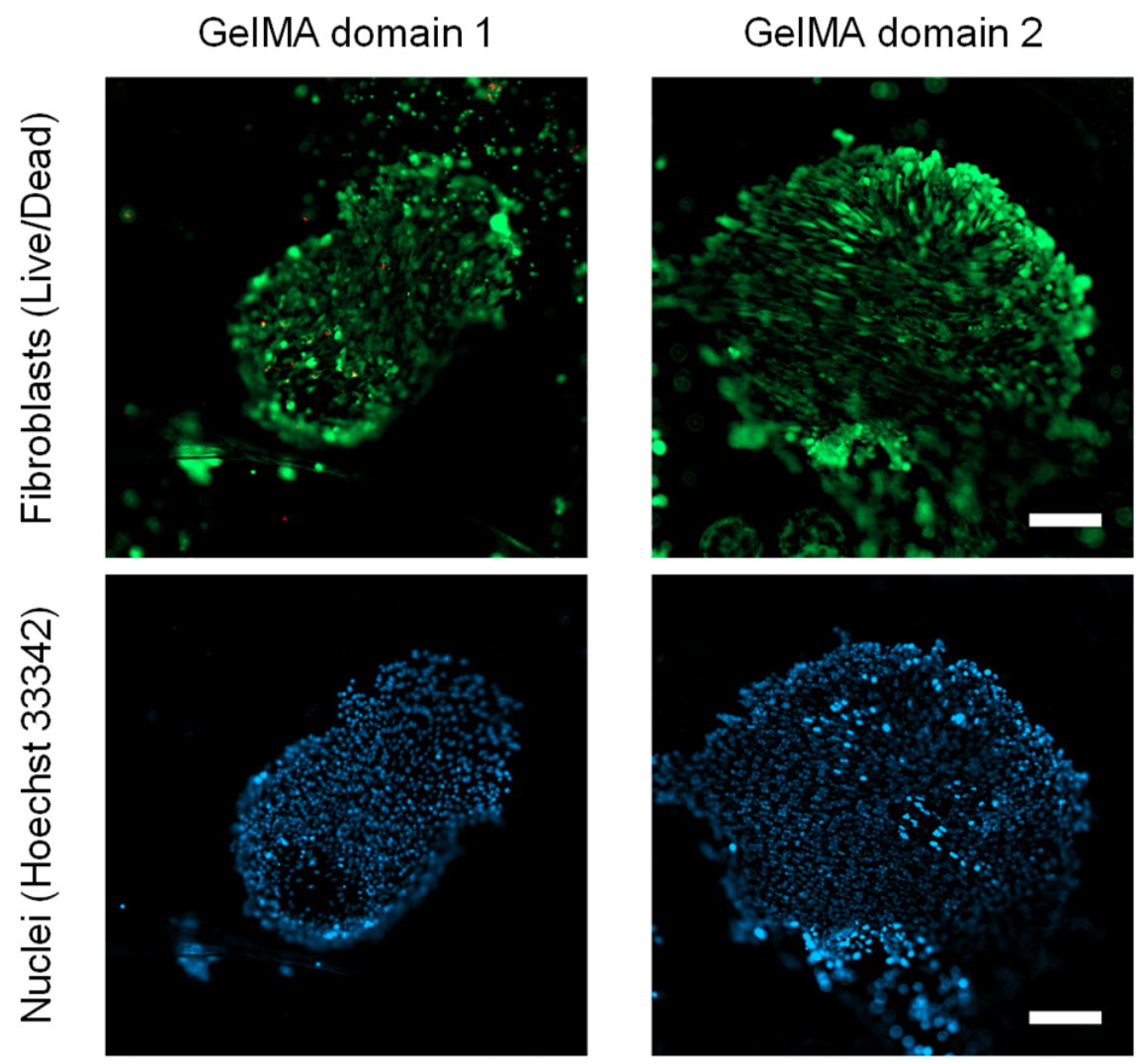

Figure S26. Cell viability in saddle constructs. Representative Live/Dead fluorescence images of fibroblasts in and around GelMA domains of saddle constructs on day 14 (top), with corresponding fluorescence images of nuclei (bottom). Across constructs, viability on day 14 was 96.8% (s.d. = 1.2%; n = 3 independent constructs). Live cells are shown in green and dead cells in red (Live/Dead assay). Nuclei are stained with Hoechst 33342 (blue). Scale bars, 200 µm.

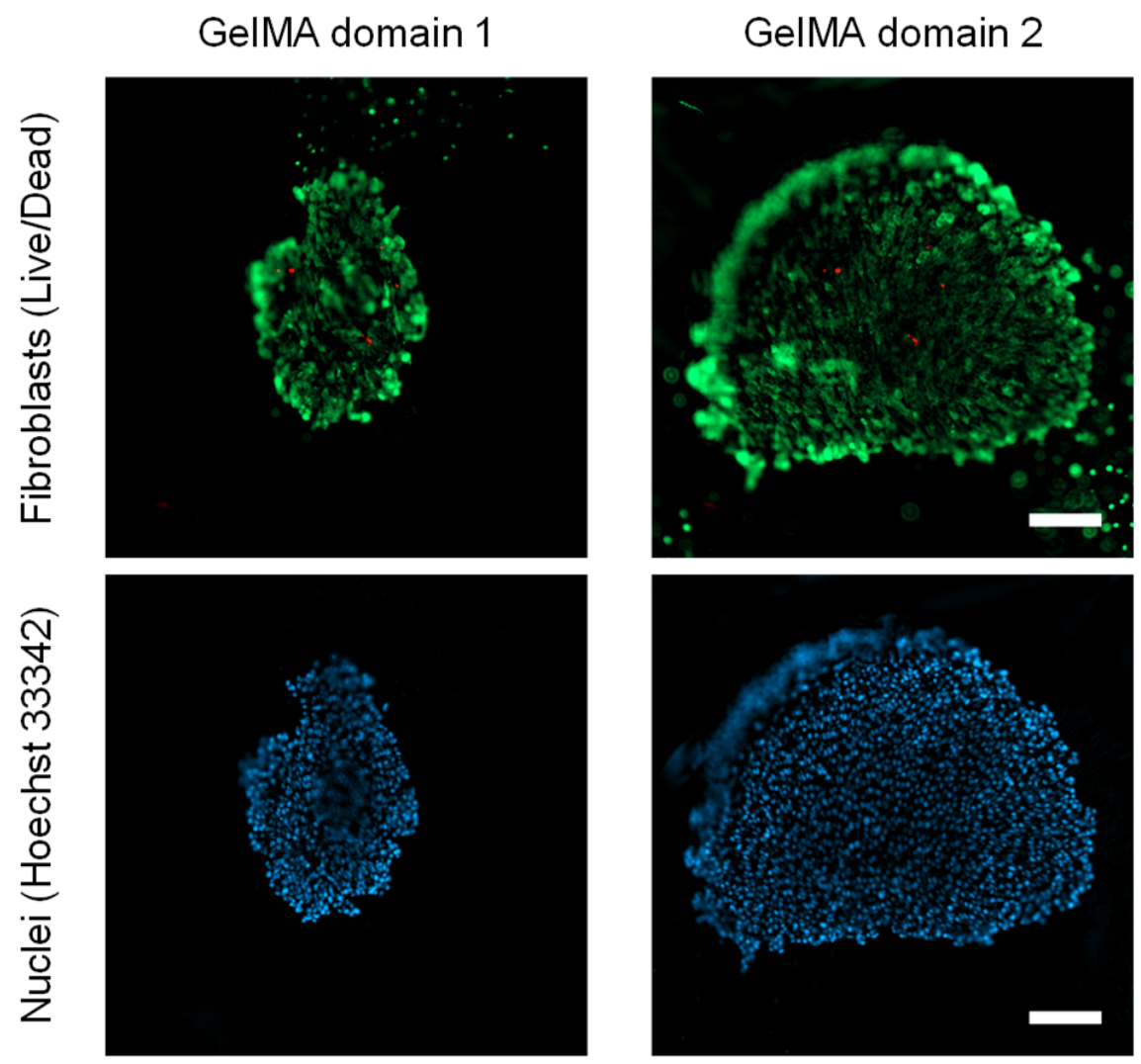

Figure S27. Cell viability in stingray-inspired constructs. Representative Live/Dead fluorescence images of fibroblasts in and around GelMA domains of stingray-inspired constructs on day 14 (top), with corresponding fluorescence images of nuclei (bottom). Across constructs, mean viability on day 14 was 94.4% (n = 2 independent constructs). Live cells are shown in green and dead cells in red (Live/Dead assay). Nuclei are stained with Hoechst 33342 (blue). Scale bars, 200 µm.

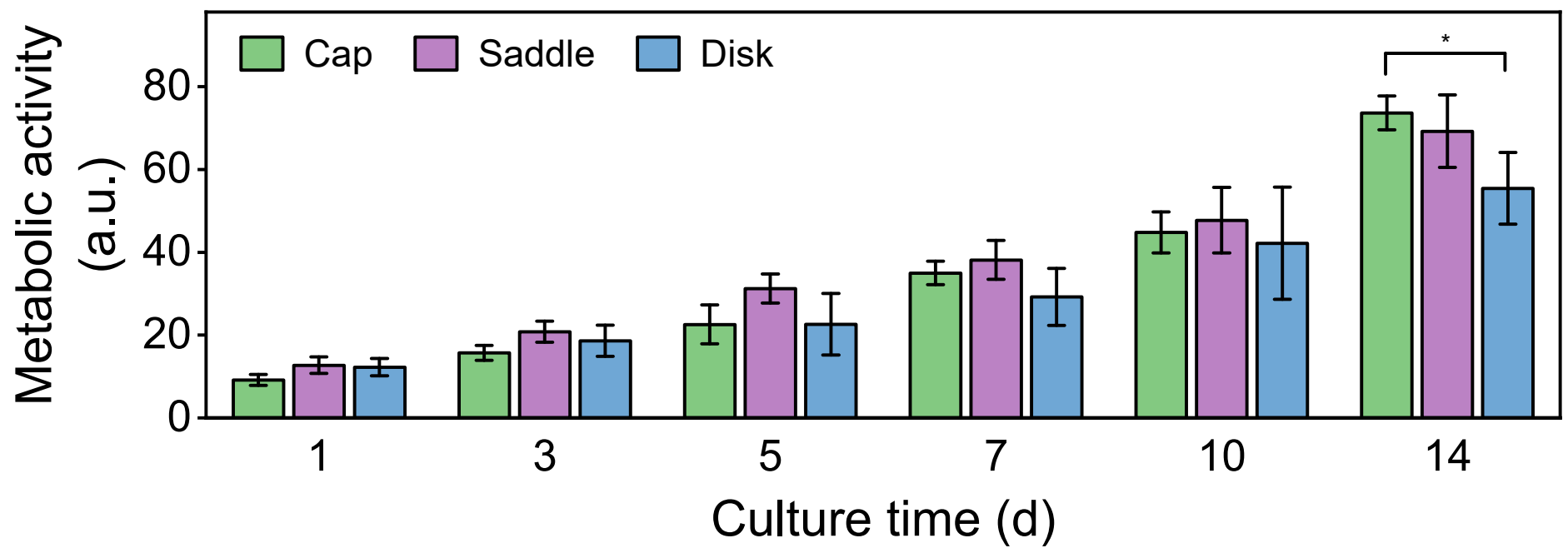

Figure S28. Metabolic activity of fibroblasts in different construct types. Metabolic activity of fibroblasts in spherical-cap constructs, saddle constructs, and non-morphing planar disk controls comprising a cell-laden GelMA domain embedded in a pNIPAm matrix. Statistics: two-way repeated-measures ANOVA with Tukey's multiple-comparisons test; n = 4 independent constructs per group, measured repeatedly over time; *P = 0.018 as indicated. All other comparisons were not significant.

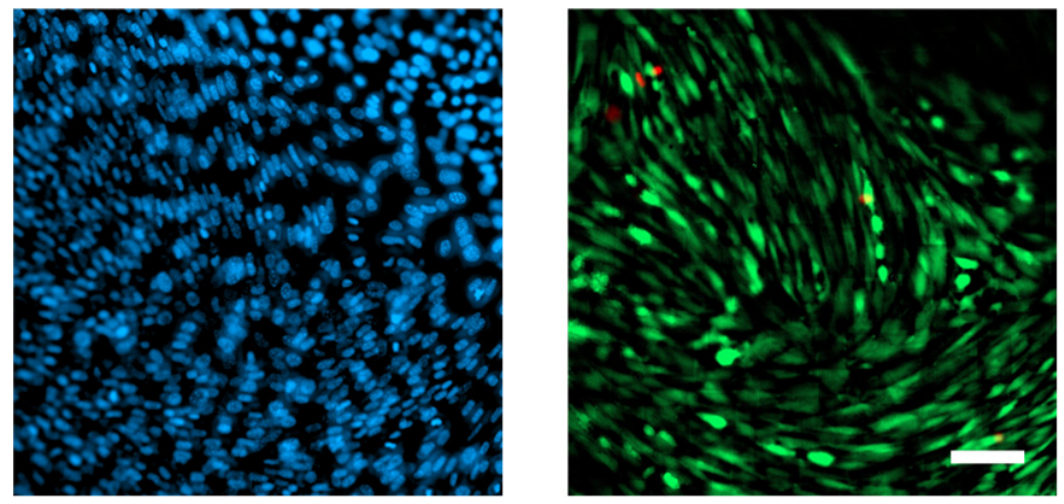

Figure S29. Representative fluorescence images of fibroblasts in a 4D-bioprinted construct. Hoechst 33342-stained nuclei (blue; left) and corresponding Live/Dead image (right) of fibroblasts in and around a GelMA domain of a spherical-cap construct on day 14. Live cells are shown in green and dead cells in red (Live/Dead assay). Scale bars, 100 µm.

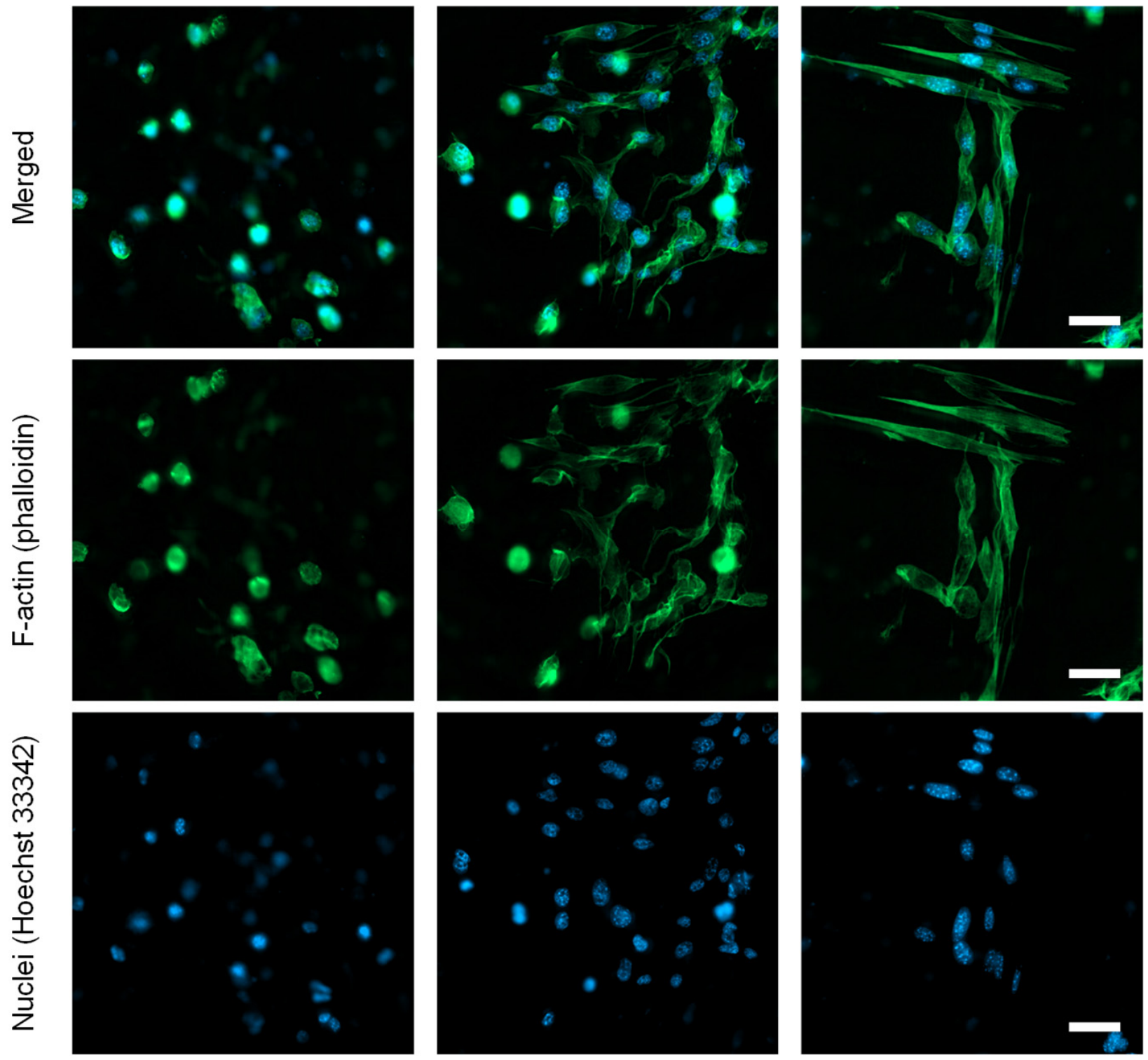

Figure S30. Representative fluorescence images of F-actin in 4D-bioprinted constructs on day 7. Merged images of F-actin (phalloidin, green) and nuclei (Hoechst 33342, blue) are shown in the top row, with corresponding single-channel F-actin (green) and nuclei (blue) images in the middle and bottom rows, respectively. Images are from spherical-cap constructs. Scale bars, 50 µm.

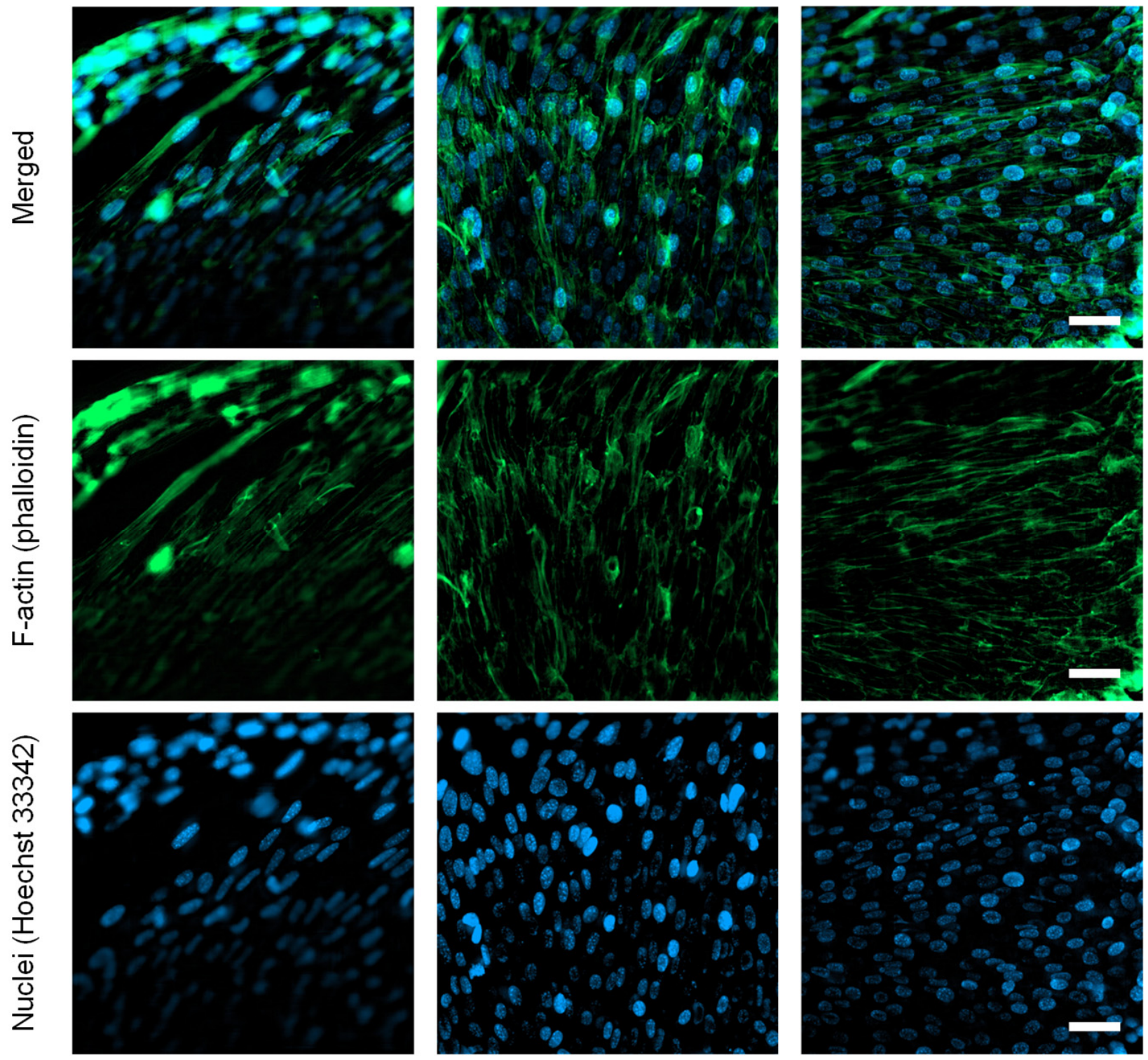

Figure S31. Representative fluorescence images of F-actin in 4D-bioprinted constructs on day 14. Merged images of F-actin (phalloidin, green) and nuclei (Hoechst 33342, blue) are shown in the top row, with corresponding single-channel F-actin (green) and nuclei (blue) images in the middle and bottom rows, respectively. Images are from spherical-cap constructs. Scale bars, 50 µm.

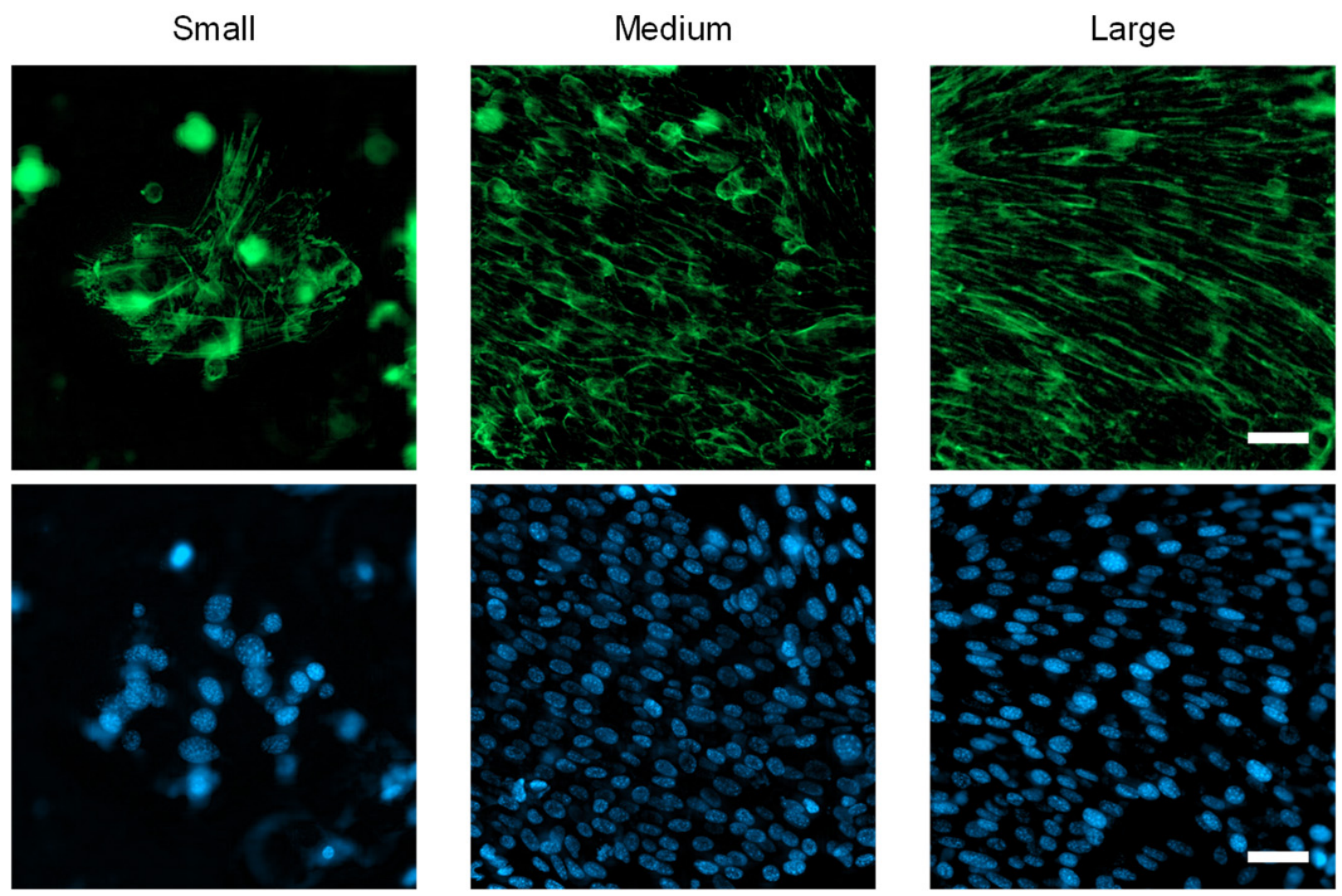

Figure S32. Representative fluorescence images of F-actin in 4D-bioprinted constructs. Phalloidin-stained F-actin (green; top row) and Hoechst 33342-stained nuclei (blue; bottom row) in small, medium, and large GelMA domains on day 14, corresponding to the merged images shown in Figure 6H. Scale bars, 50 µm.

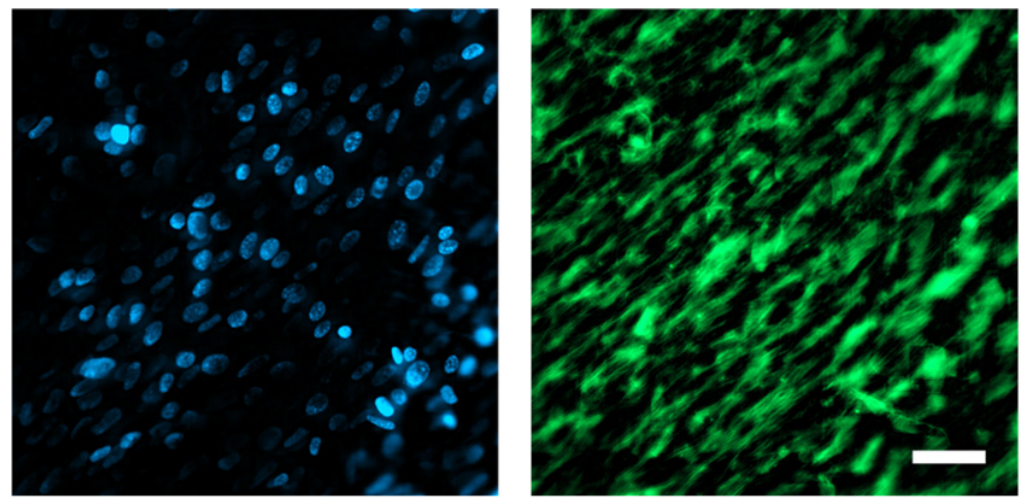

Figure S33. Representative fluorescence images of F-actin in a 4D-bioprinted saddle construct. Hoechst 33342-stained nuclei (blue; left) and phalloidin-stained F-actin (green; right) on day 14. Scale bars, 50 µm.

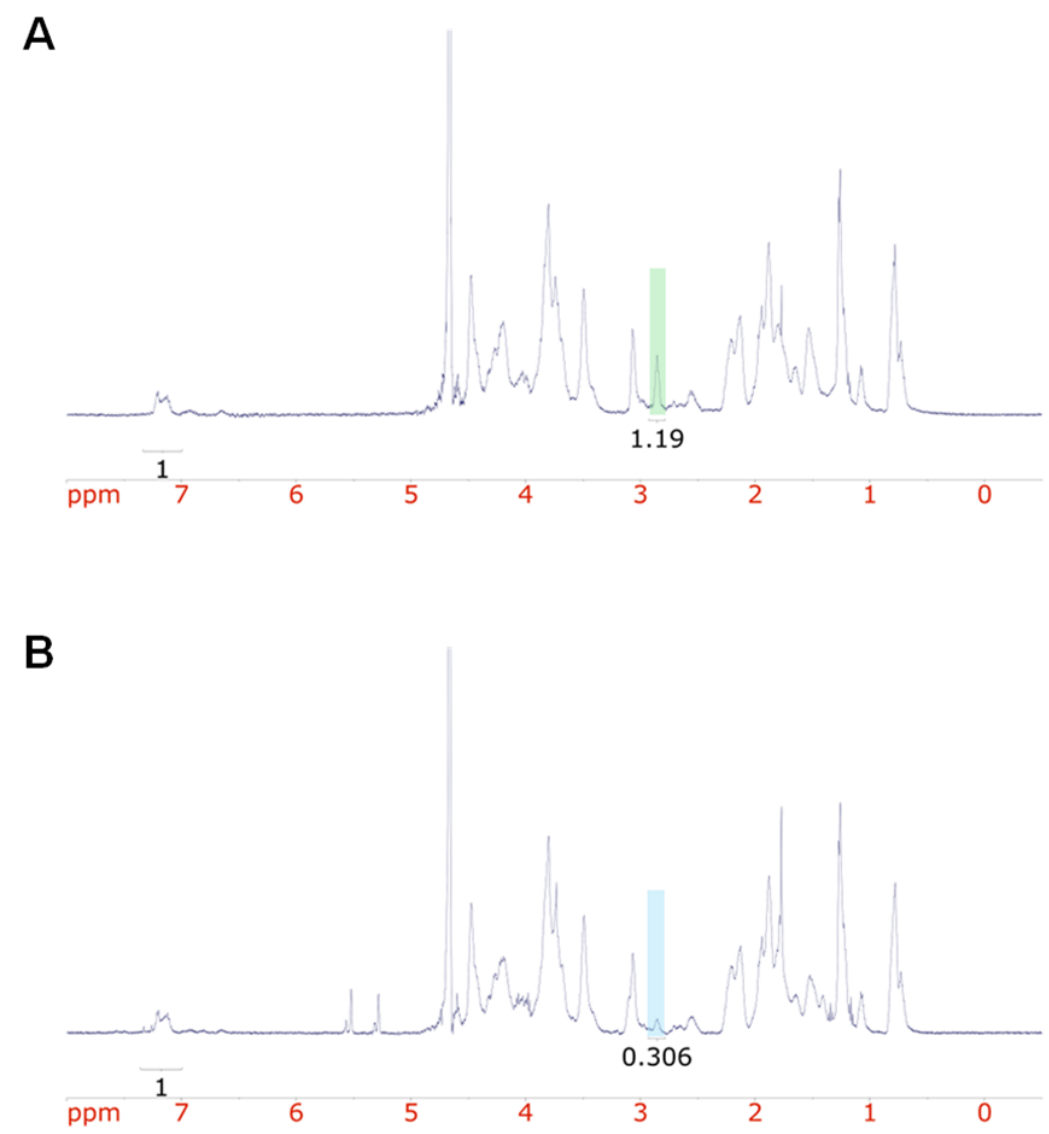

Figure S34. ^{1}H NMR spectra of gelatin and GelMA in D_2O. (A) ^{1}H NMR spectrum of gelatin. (B) ^{1}H NMR spectrum of GelMA with a degree of methacryloyl modification of 74.3%. The degree of modification was quantified from the lysine methylene signals in the gelatin and GelMA spectra, highlighted in green (A) and blue (B), respectively [4, 5].

Table S1. Principal curvatures of hybrid constructs in Figure 3C and Figure S9

	Theoretical k (mm^{-1})	Experimentally measured k (mm^{-1})		
		Construct with cuts (Figure S9A)	Construct with linkers (Figure S9B)	Construct without cuts or linkers (Figure S9C)
$K > 0$ domain	0.24	0.24	0.23	0.23
$K < 0$ domain	−0.3	−0.31	−0.26	−0.25

* The principal curvatures k were measured in the $K > 0$ and $K < 0$ domains from the side views of the hybrid constructs, viewed toward one of the $K > 0$ and $K < 0$ interfaces as shown in Figure S9.

Movie S1. Reversible shape transformation of a spherical-cap structure. Reversible transformation between the shrunken and swollen states. Shown at 450× real time.

Movie S2. Reversible shape transformation of a saddle structure. Reversible transformation between the shrunken and swollen states. Shown at 450× real time.

Movie S3. Formation of a stingray-inspired construct (example 1). Shape transformation on a substrate from a configuration in the swollen state to the target 3D shape in the shrunken state. Shown at 900× real time.

Movie S4. Formation of a stingray-inspired construct (example 2). Shape transformation on a substrate from a configuration in the swollen state to the target 3D shape in the shrunken state. Shown at 900× real time.

Movie S5. Formation of a stingray-inspired construct (example 3). Shape transformation on a pin from a configuration in the swollen state to the target 3D shape in the shrunken state. Shown at 900× real time.

Movie S6. Reversible shape transformation of a stingray-inspired construct. Reversible transformation between the shrunken and swollen states. Shown at 900× real time.

Movie S7. Oscillatory flapping motion of a stingray-inspired construct. Oscillatory flapping under cyclic temperature modulation. Shown at 700× real time.

References

1. J. Kim, J. A. Hanna, M. Byun, C. D. Santangelo, R. C. Hayward, “Designing Responsive Buckled Surfaces by Halftone Gel Lithography,” *Science* **335** (2012): 1201-1205.
2. A. Nojoomi, H. Arslan, K. Lee, K. Yum, “Bioinspired 3D Structures with Programmable Morphologies and Motions,” *Nature Communications* **9** (2018): 3705.
3. A. Nojoomi, J. Jeon, K. Yum, “2D Material Programming for 3D Shaping,” *Nature Communications* **12** (2021): 603.
4. H. Arslan, A. Davuluri, H. H. Nguyen et al., “3D Bioprinting Using Universal Fugitive Network Bioinks,” *ACS Applied Bio Materials* **7** (2024): 7040-7050.
5. E. Hoch, T. Hirth, G. E. M. Tovar, K. Borchers, “Chemical Tailoring of Gelatin to Adjust Its Chemical and Physical Properties for Functional Bioprinting,” *Journal of Materials Chemistry B* **1** (2013): 5675-5685.
